# CMPK2 restricts Zika virus replication by inhibiting viral translation

**DOI:** 10.1371/journal.ppat.1011286

**Published:** 2023-04-19

**Authors:** Joanna B. Pawlak, Jack Chun-Chieh Hsu, Hongjie Xia, Patrick Han, Hee-Won Suh, Tyler L. Grove, Juliet Morrison, Pei-Yong Shi, Peter Cresswell, Maudry Laurent-Rolle

**Affiliations:** 1 Department of Immunobiology, Yale University School of Medicine, New Haven, Connecticut, United States of America; 2 Section of Infectious Diseases, Department of Internal Medicine, Yale University School of Medicine, New Haven, Connecticut, United States of America; 3 Department of Microbial Pathogenesis, Yale University School of Medicine, New Haven, Connecticut, United States of America; 4 Department of Biochemistry and Molecular Biology, University of Texas Medical Branch, Galveston, Texas, United States of America; 5 Department of Dermatology, Yale University School of Medicine, New Haven, Connecticut, United States of America; 6 Department of Biomedical Engineering, Yale University School of Engineering and Applied Science, New Haven, Connecticut, United States of America; 7 Department of Biochemistry, Albert Einstein College of Medicine, Bronx, New York, United States of America; 8 Department of Microbiology and Plant Pathology, University of California, Riverside, California, United States of America; 9 Institute for Human Infections and Immunity, University of Texas Medical Branch, Galveston, Texas, United States of America; 10 Sealy Institute for Vaccine Sciences, University of Texas Medical Branch, Galveston, Texas, United States of America; 11 Sealy Institute for Drug Discovery, University of Texas Medical Branch, Galveston, Texas, United States of America; 12 Department of Cell Biology, Yale University School of Medicine, New Haven, Connecticut, United States of America; UT Southwestern: The University of Texas Southwestern Medical Center, UNITED STATES

## Abstract

Flaviviruses continue to emerge as global health threats. There are currently no Food and Drug Administration (FDA) approved antiviral treatments for flaviviral infections. Therefore, there is a pressing need to identify host and viral factors that can be targeted for effective therapeutic intervention. Type I interferon (IFN-I) production in response to microbial products is one of the host’s first line of defense against invading pathogens. Cytidine/uridine monophosphate kinase 2 (CMPK2) is a type I interferon-stimulated gene (ISG) that exerts antiviral effects. However, the molecular mechanism by which CMPK2 inhibits viral replication is unclear. Here, we report that CMPK2 expression restricts Zika virus (ZIKV) replication by specifically inhibiting viral translation and that IFN-I- induced CMPK2 contributes significantly to the overall antiviral response against ZIKV. We demonstrate that expression of CMPK2 results in a significant decrease in the replication of other pathogenic flaviviruses including dengue virus (DENV-2), Kunjin virus (KUNV) and yellow fever virus (YFV). Importantly, we determine that the N-terminal domain (NTD) of CMPK2, which lacks kinase activity, is sufficient to restrict viral translation. Thus, its kinase function is not required for CMPK2’s antiviral activity. Furthermore, we identify seven conserved cysteine residues within the NTD as critical for CMPK2 antiviral activity. Thus, these residues may form an unknown functional site in the NTD of CMPK2 contributing to its antiviral function. Finally, we show that mitochondrial localization of CMPK2 is required for its antiviral effects. Given its broad antiviral activity against flaviviruses, CMPK2 is a promising potential pan-flavivirus inhibitor.

## Author summary

Flaviviruses are arthropod-borne viruses for which there are no effective therapeutic agents. Therefore, there is an urgent need to develop new antivirals and vaccines. Our study characterizes CMPK2 as a flaviviral restriction factor that exerts its antiviral activity by specifically inhibiting viral translation. We demonstrate that CMPK2 induction significantly decreases the replication of dengue (DENV-2), Zika (ZIKV), Kunjin (KUNV) and yellow fever (YFV) viruses. We show that the antiviral function of CMPK2 depends on its entry into the mitochondria but is independent of the C-terminal kinase domain. Thus, the N-terminal domain of CMPK2 is sufficient to inhibit Zika virus translation. These findings provide a basis for the development of new antivirals against flaviviruses focused on CMPK2 as a potent inhibitor.

## Introduction

Flaviviruses belong to the *Flaviviridae* family and are positive-sense, single-stranded RNA viruses. Flaviviruses are primarily transmitted to humans by infected arthropods with clinical manifestations ranging from an acute self-limiting febrile disease to severe illnesses and death [1]. Flaviviruses, including dengue virus (DENV), West Nile virus (WNV), yellow fever virus (YFV), Powassan virus (POWV), Japanese encephalitis virus (JEV) and Zika virus (ZIKV) are medically relevant pathogens and continue to pose a threat globally. Increased travel and population growth, combined with failed vector control programs have led to the spread of flaviviruses beyond their geographical borders. Worldwide, about 400 million people are infected with DENV annually, resulting in several hundred thousand cases of dengue hemorrhagic fever and thousands of deaths [1,2]. WNV infections have caused more than 2,600 deaths in the United States since its introduction in the United States in 1999 [3,4]. Despite the availability of a highly efficacious YFV vaccine (YFV17D), there was a resurgence of yellow fever in Brazil between 2016 and 2019 with a 35% fatality ratio for severe yellow fever cases, resulting in nearly 800 deaths [5–7]. In addition, the ZIKV epidemic in Latin America and the Caribbean in 2015, with its association with congenital microcephaly and other developmental defects in infants born to infected women, underscores the need for development of antivirals to combat flaviviruses [8].

The type I interferon (IFN-I) system is the host’s first line of defense against infectious agents. Upon viral infection, pattern recognition receptors (PRRs) like the Toll-like receptors (TLRs) and retinoic acid-inducible gene I (RIG-I) and melanoma differentiation-associated protein 5 (MDA5) sense viral nucleic acids, activating downstream signaling factors that ultimately result in the production of IFN-I. Secreted IFN-I signals in an autocrine and paracrine manner by binding to IFN-I receptors on cell surfaces. Binding to the IFN-I receptors leads to the downstream activation of the JAK-STAT signaling pathway, which eventually results in protein expression of IFN-stimulated genes (ISGs), including *CMPK2*, leading to an antiviral state to curb viral replication and dissemination [9–11]. There are a variety of mechanisms by which those ISGs restrict viral infection by inhibiting different stages of the virus life cycle. Since viral replication heavily depends on the host’s protein synthesis machinery, a common response is to suppress global and/or viral translation [12,13]. For example, some ISGs induce RNA degradation [14,15] or the integrated stress response (ISR) [16,17] to inhibit viral translation.

While use of IFN-I itself as an antiviral agent is associated with adverse effects and high cost, targeting individual ISGs and unraveling their *modus operandi* during the antiviral response is of critical importance to develop effective antiviral therapies. The *CMPK2* gene encodes the interferon-stimulated protein, cytidylate monophosphate kinase 2 (CMPK2), which is highly conserved in mammals (S1 Fig). In mammalian genomes, the *CMPK2* gene is immediately adjacent to *RSAD2*, the gene encoding an antiviral protein, viperin, which is co-transcribed with CMPK2 during cellular stimulation with IFN, lipopolysaccharide (LPS) and polycytidylic acid (poly (I:C)), suggesting a functional linkage between CMPK2 and viperin [18,19].

**Fig 1:**
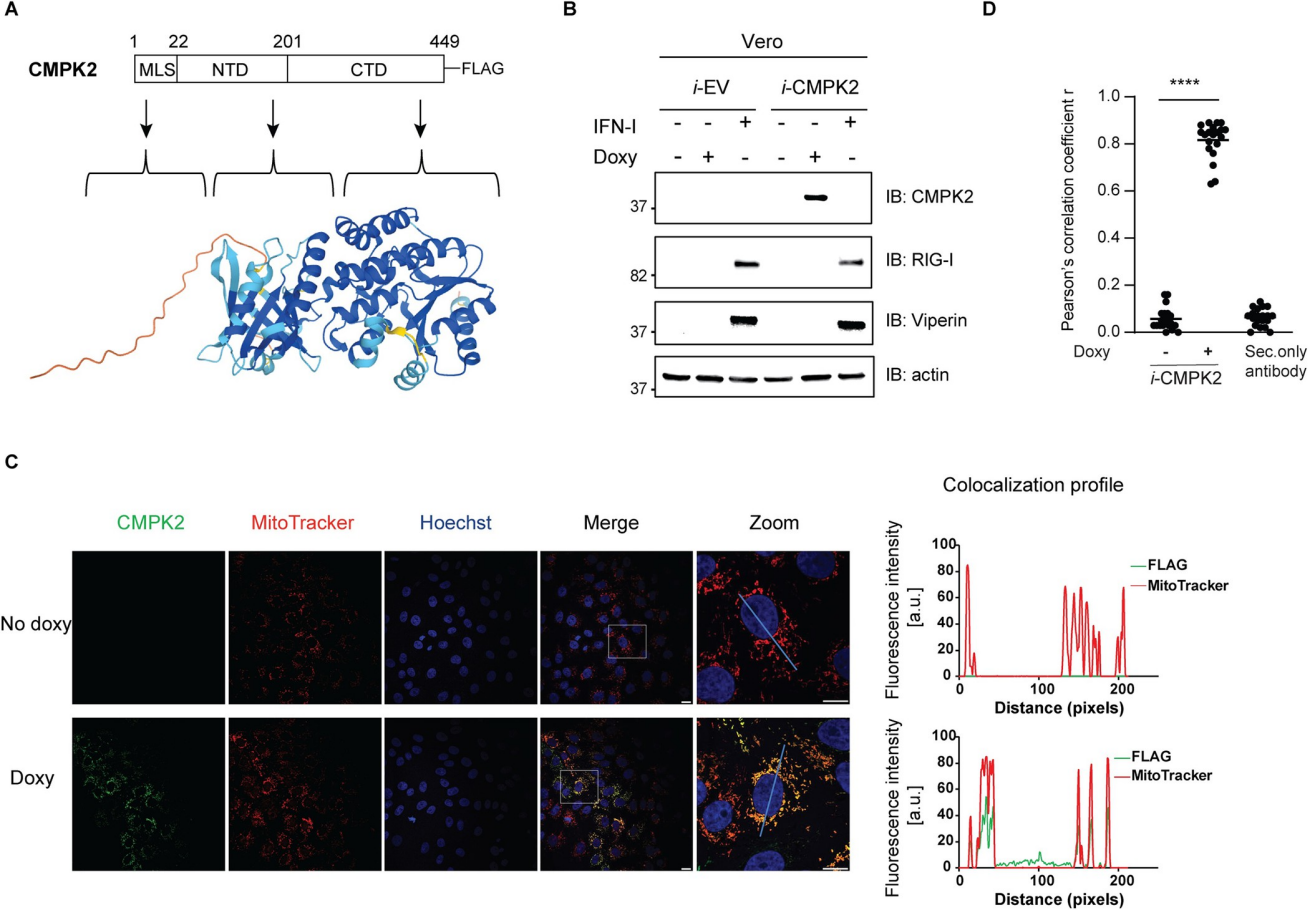
Characterization of doxycycline-inducible Vero i-CMPK2 cells. **(A)** Schematic illustration of the FLAG-tagged CMPK2 construct used in Fig 1 (upper panel). AlphaFold predicted human CMPK2 structure as shown in the lower panel [50]. **(B)** Immunoblot analysis of Vero *i*-EV and *i*-CMPK2 cells that were mock- or doxycycline-treated or IFN-I-treated for 24 h. Cell lysates were analyzed by immunoblotting with anti-CMPK2, anti-RIG-I, anti-viperin and anti-actin antibodies. Doxy = doxycycline. **(C)** Immunofluorescence analysis of Vero *i-*CMPK2 cells that were mock- or doxycycline-treated for 24 h, stained with MitoTracker (a mitochondrial marker), followed by fixation, permeabilization, and intracellular staining with anti-FLAG antibody for FLAG-tagged CMPK2. Colocalization of FLAG with MitoTracker is shown in rectangles (zoom) and colocalization profiles are on the right. All images were processed identically using the same settings and colocalization (RGB) profiles were obtained using ImageJ (NIH). Secondary only control in S3 Fig. a.u. = arbitrary unit. **(D)** Pearson’s correlation coefficient r for FLAG and MitoTracker fluorescence determined in mock- and doxycycline-treated *i*-CMPK2 cells from panel C. Black horizontal bars represent the mean of the group (n = 20). ****p < 0.0001 by unpaired Student’s t test.

The expression of CMPK2 in mammals is distributed ubiquitously across various tissues [20]. High levels of CMPK2 are observed in myeloid, lymphoid and mesenchymal tissues [21,22]. Human CMPK2 is a 449-amino-acid long protein that can be separated into two domains, an N-terminal domain (NTD, amino acids 23–201) with a mitochondrial localization sequence (MLS, amino acid residues 1–22) that primarily localizes it to the mitochondria, and a C-terminal domain (CTD, amino acid residues 202–449) (Fig 1A) [23]. The CTD of CMPK2 is highly conserved across species and is responsible for its kinase activity (S1 Fig) [23]. CMPK2 was initially reported to belong to a novel nucleoside monophosphate kinase family [23]. However, more recently, CMPK2’s kinase domain was reported to preferentially phosphorylate cytidine diphosphate (CDP) and uridine diphosphate (UDP) to cytidine triphosphate (CTP) and uridine triphosphate (UTP), respectively [18]. CMPK2 kinase activity plays a pivotal role in antiviral responses. The kinase activity functions as a CTP provider for viperin. Viperin uses CTP as a substrate to synthesize 3’-deoxy-3’,4’-didehydro-cytidine triphosphate (ddhCTP) during the IFN-I response [18]. ddhCTP was shown to be incorporated into nascent RNA and to act as a chain terminator for the RNA-dependent RNA polymerases of multiple flaviviruses *in vitro* and directly inhibit ZIKV replication *in vivo* [18]. However, recently, we reported that viperin’s enzymatic activity and its product ddhCTP are required to restrict global and viral translation through enhanced ribosome collisions [17]. The kinase activity of CMPK2 was also demonstrated to be required for inflammasome activation [24]. Given the low sequence homology to any other known proteins, the molecular/biological function of the NTD of CMPK2 is unknown. Protein sequence analysis shows that the NTD contains nine cysteine residues, six of which are conserved across vertebrate CMPK2s (S1 Fig). A high number of cysteine residues typically indicates either disulfide bonds, metal binding [25] or enzyme catalytic activity [23,26]. CMPK2 may work in synergy with viperin to establish an antiviral state, however, whether CMPK2 antiviral activity is dependent or independent of viperin has not been extensively investigated.

CMPK2 has been reported to have an additional function as an antiviral protein against several viruses. A recent study showed that CD4+ T cells from patients co-infected with human immunodeficiency virus (HIV) and hepatitis C virus (HCV) that were treated with IFN-α resulted in induction of multiple ISGs including CMPK2 [27]. The antiviral effect of IFN on HIV replication was attenuated in a human monocytic cell line (THP-1 cells) with reduced expression of CMPK2, resulting in a significant increase in HIV viral titers which suggests that CMPK2 has antiviral activity against HIV [27]. CMPK2 was also reported to inhibit DENV replication. Knockdown of CMPK2 in mouse bone marrow derived macrophages and dendritic cells as well as in human monocyte-derived dendritic cells resulted in an increase in DENV RNA, while overexpression of CMPK2 in BMDCs and the lung epithelial cell line A549 resulted in a decrease in DENV RNA [21]. In zebrafish, overexpression of CMPK2 resulted in a decrease of spring viremia of carp virus (SVCV) while knockdown of CMPK2 in SVCV-infected cells showed a significant increase in SVCV demonstrating an antiviral effect against SVCV infection [28]. Chicken CMPK2 (chCMPK2) was reported to have antiviral activity against Avian influenza and Newcastle disease virus infection in chicken fibroblasts [29]. However, the mechanism behind the antiviral activity of CMPK2 was not elucidated in these studies and it was not reported if viperin plays a role in CMPK2’s antiviral activity. We examined the breadth of CMPK2’s antiviral activity against viruses from various families, including *Flaviviridae*, *Coronaviridae*, *Orthomyxoviridae*, *Paramyxoviridae* and *Herpesviridae* by CMPK2 overexpression as well as CRISPR/Cas9-mediated CMPK2 knockout studies in human and non-human fibroblasts. We show that CMPK2 restricts the replication of multiple, medically relevant flaviviruses: DENV serotype 2 (DENV-2); ZIKV; Kunjin virus (KUNV), which is a subtype of WNV; and YFV17D, a YFV vaccine strain. However, CMPK2 expression had no effect on the replication of other RNA viruses, including influenza A virus (IAV) and severe acute respiratory syndrome coronavirus 2 (SARS-CoV-2). We show that CMPK2 restricts ZIKV replication by specifically inhibiting virus translation and that its mitochondrial localization is required. In addition, our data show that the NTD of CMPK2 is sufficient to inhibit the replication of ZIKV. There is great interest in developing new antiviral therapies based on ISG functions rather than targeting viral proteins to minimize the emergence of antiviral drug resistance. Given its activity against multiple members of the *Flaviviridae* virus family, CMPK2 may provide an effective focus for developing specific therapies against flaviviruses.

## Results

### CMPK2 is required to inhibit Zika virus in the type I interferon response

**Fig 2:**
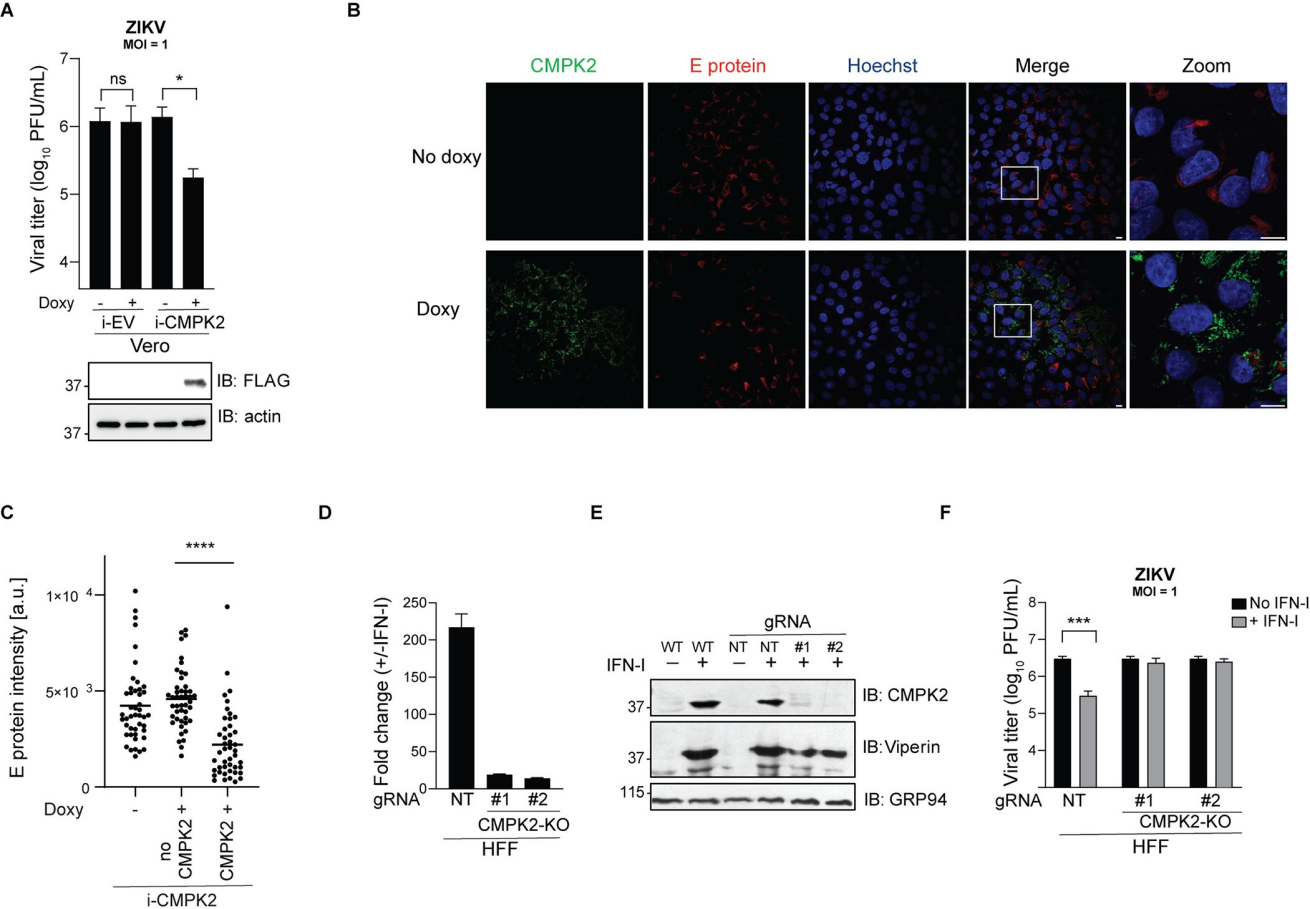
CMPK2 inhibits Zika virus replication. **(A)** Quantification of ZIKV production in Vero *i*-EV and *i*-CMPK2 cells that were mock- or doxycycline-treated for 24 h and infected with ZIKV at an MOI of 1 for 24 h. Viral titers were determined by plaque assay. Cell lysates were analyzed by immunoblotting with anti-FLAG and anti-GRP94 antibodies. Data are shown as mean ± SD of two biological repeats (n = 2). ns = not significant; *p < 0.05 by unpaired Student’s t test. MOI = multiplicity of infection; PFU = plaque forming units. **(B)** Immunofluorescence analysis of the ZIKV E proteins in Vero *i*-CMPK2 cells. Cells were mock- or doxycycline-treated for 24 h then infected with ZIKV at an MOI of 0.1. 48 hpi cells were fixed, permeabilized and stained by anti-E protein and anti-FLAG antibodies and analyzed by confocal immunofluorescence microscopy. Uninfected control in S4 Fig. MOI = multiplicity of infection; hpi = hours post infection. **(C)** Quantification of the ZIKV E protein fluorescence intensities in Vero *i*-CMPK2 cells from panel B. Total E protein intensity is quantified as (cell area* mean E protein intensity). Black horizontal bars represent the mean of the group (n = 45). ****p < 0.0001 by unpaired Student’s t test. No CMPK2 = doxycycline-treated but no CMPK2 expression present. a.u. = arbitrary unit. **(D)** qRT-PCR analysis of *CMPK2* RNA in NT and CMPK2-KO (#1 and #2) HFF cells. Data are shown as mean ± SD of two biological repeats (n = 2). NT = non-targeting; KO = knockout. **(E)** Immunoblot analysis of CMPK2 expression in WT, NT and CMPK2-KO (#1 and #2) HFF cells. Cells were mock or IFN-I treated for 24 h, then lysed and analyzed by immunoblotting with anti-CMPK2, anti-viperin, and anti-GRP94 antibodies. WT = wild-type. **(F)** Quantification of ZIKV production in NT or CMPK2-KO HFF cells. Cells were infected with ZIKV at an MOI of 1. 6 hpi, cells were mock or treated with 1,000 U/mL universal IFN-I. 24 hpi, supernatants were analyzed by plaque assay. Data are shown as mean ± SD of three biological repeats (n = 3). ***p < 0.001 by unpaired Student’s t test. hpi = hours post infection.

Human CMPK2 suppresses replication of HIV-I and DENV, which are both single-stranded, positive-sense RNA viruses [21,27]. Given the similarities in the biochemistry and molecular biology of the flaviviruses, we assessed whether CMPK2 exhibits an antiviral effect against other medically relevant flaviviruses, initially ZIKV. To evaluate its biological effects, we generated a doxycycline-inducible Vero E6 cell line in which CMPK2 expression could be induced in a dose- and time-dependent manner. We used Vero E6 cells as the background cell line for the doxycycline-inducible system because Vero E6 cells have a genetic defect in IFN-I production but respond to exogenous IFN-I, ensuring that the inducible protein will only be expressed upon doxycycline treatment [30,31]. Vero E6 cells were transduced with either a lentivirus encoding empty vector control (Vero *i*-EV) or CMPK2 bearing a C-terminal FLAG tag (Vero *i*-CMPK2) (Fig 1A). We validated the stringency of the doxycycline-inducible system by measuring *CMPK2* RNA levels by qRT-PCR. A substantial increase in *CMPK2* RNA was observed in the doxycycline- and IFN-I-treated Vero *i*-CMPK2 cells (S2A Fig). We also observed an increase in *CMPK2* RNA in IFN-I treated Vero *i*-EV cells, but not in mock- or doxycycline-treated Vero *i*-EV cells (S2A Fig). In addition, we used the well characterized IFN-stimulated *RSAD2* gene, which encodes viperin, as a control. A robust increase in *RSAD2* RNA was observed only upon addition of IFN-I (S2B Fig). Next, we verified CMPK2 expression by immunoblot analysis. We noted that CMPK2 was expressed only in Vero *i*-CMPK2 cells treated with doxycycline (Fig 1B). Moreover, the IFN-inducible proteins; viperin and RIG-I were detected only upon treatment with IFN-I (Fig 1B). These results suggested that Vero *i*-CMPK2 cells are suitable to study CMPK2 in the absence of other ISG-encoded proteins. Note in Fig 1B, we were unable to detect IFN-I induced endogenous CMPK2 in African green monkey Vero E6 cell lines because our antibody was generated using an immunogenic peptide sequence that differs between human and non-human primates. Next, we confirmed the subcellular localization of CMPK2 by confocal microscopy, finding that it was expressed in doxycycline-treated cells, and as expected, given its MLS, primarily localized to the mitochondria (Fig 1C) [23]. Indeed, the CMPK2 expression correlated with the mitochondrial marker (MitoTracker) intensities (Figs 1C, 1D and S3). To determine whether CMPK2 expression inhibits ZIKV replication, Vero *i*-EV and *i*-CMPK2 cells were mock- or doxycycline-treated, ZIKV infected at a multiplicity of infection (MOI) of 1 and viral titers were determined by plaque assay. We observed that ZIKV replication was significantly inhibited by the expression of CMPK2 compared to mock-treated Vero *i*-CMPK2 cells (Fig 2A). Moreover, using immunofluorescence analysis, we showed that cells expressing CMPK2 had significantly less viral envelope (E) protein staining, as compared to non-CMPK2-expressing cells (Figs 2B, 2C and S4). Together, these results indicated that CMPK2 inhibits ZIKV replication.

To complement the overexpression studies, we used CRISPR/Cas9 to knockout CMPK2 in an immortalized human foreskin fibroblast cell line (HFF) [32]. For this experiment, we chose to use HFF cells since endogenous CMPK2 is readily induced upon IFN-I treatment and can be detected by our human CMPK2 antibody [32,33]. A GFP-selectable lentiCRISPR vector was used to deliver a nontargeting gRNA (NT) as a control and gRNAs (#1 and #2) targeting *CMPK2* to the cells. Loss of IFN-I-induced CMPK2 gene expression in the CMPK2-KO HFF cells was determined by qRT-PCR (Fig 2D) and loss of protein expression was confirmed by Western blotting (Fig 2E). In the absence of IFN-I, ZIKV replicated to similar titers in NT and CMPK2-KO HFF cells. IFN-I treatment suppressed ZIKV replication significantly in NT HFF cells at 24 hours post infection (hpi), but notably there was no decrease in ZIKV titers in IFN-I-treated CMPK2-KO HFF cells, suggesting that CMPK2 is required to facilitate IFN-I-mediated restriction of ZIKV replication (Fig 2F). This may be partially explained by the functional linkage of CMPK2 and viperin wherein the kinase domain of CMPK2 provides the CTP substrate for viperin [18]. Thus, in the absence of CMPK2, viperin may be unable to affect ZIKV replication due to suboptimal levels of CTP substrate to produce enough ddhCTP [17,18]. Thus, loss of CMPK2 activity may dampen the antiviral activity of two components known to affect flavivirus replication. These results are consistent with published data by El. Diwany *et al*. who showed that CMPK2 plays a significant role in the IFN response to HIV despite the upregulation of other ISGs including the well characterized *RSAD2* (viperin) which has been reported to restrict HIV [27,34]. Taken together, these results implicate CMPK2 as an important host restriction factor of ZIKV.

### CMPK2 inhibits the replication of multiple pathogenic flaviviruses

**Fig 3:**
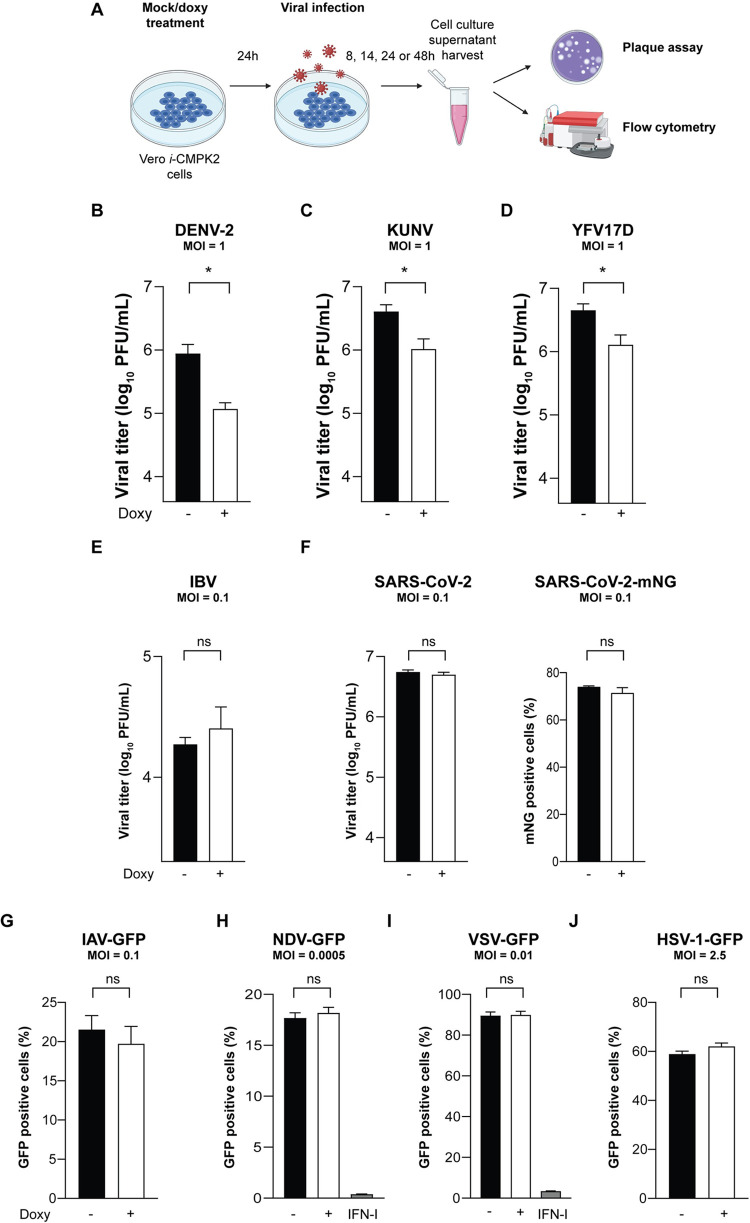
CMPK2 inhibits the replication of multiple pathogenic flaviviruses but not viruses of the *Corona-*, *Orthomyxo-*, *Paramyxo-*, *Rhabdo-* and *Herpes-viridae* families. **(A)** Quantification of viral production in Vero *i*-CMPK2 cells. Cells were mock-, doxycycline- or IFN-I treated for 24 h and then infected with **(B)** dengue virus serotype 2 (DENV-2), **(C)** Kunjin virus (KUNV) or **(D)** yellow fever virus vaccine strain (YFV17D) at an MOI of 1 for 24 h or for 48 h for DENV-2, and viral titers were determined by plaque assay. **(E)** avian infectious bronchitis virus (IBV) or **(F)** severe acute respiratory syndrome coronavirus 2 (SARS-CoV-2) and SARS-CoV-2 expressing mNeonGreen fluorescent protein (mNG) at an MOI of 0.1 for 24 h, and viral titers were determined by plaque assay or flow cytometry **(A)**. **(G-J)** Quantification of IAV, NDV, VSV and HSV-1 replication in Vero *i*-CMPK2 cells. Cells were mock- or doxycycline-treated for 24 h and then infected with green fluorescent protein (GFP) reporter viruses: **(G)** influenza A virus (IAV-GFP) at an MOI of 0.1 for 24 h, **(H)** Newcastle disease virus (NDV-GFP) at an MOI of 0.0005 for 14 h, **(I)** vesicular stomatitis virus (VSV-GFP) at an MOI of 0.01 for 24 h, or **(J)** herpes simplex type 1 virus (HSV-1-GFP) at an MOI of 2.5 for 8 h; GFP-positive cells were determined by flow cytometry. Data are shown as mean ± SD of three biological repeats (n = 3). *p < 0.05, ns = not significant by unpaired Student’s t test. MOI = multiplicity of infection; PFU = plaque forming unit; Doxy = doxycycline. IFN-I = Interferon type I. Illustration in (A) was created with BioRender.com.

Generation of novel broad-spectrum antivirals is needed to prepare us for inevitable viral epidemics and pandemics. Since CMPK2 has an antiviral effect against ZIKV (Fig 2A) and was recently reported to inhibit DENV replication [21], we examined its activity against DENV-2 and other pathogenic flaviviruses, given that the flaviviruses are closely related [1]. We mock- or doxycycline-treated Vero *i*-CMPK2 cells for 24 hours, then infected them with DENV-2 at an MOI of 1 for 48 hours, and determined viral titers by plaque assay (Fig 3A). We showed that CMPK2 significantly inhibits the replication of DENV-2 (Fig 3B). We also examined CMPK2’s antiviral effect on KUNV infection, which in humans mostly causes a mild febrile illness although some individuals can develop encephalitis [35,36]. Vero *i*-CMPK2 cells were mock- or doxycycline-treated, infected with KUNV at an MOI of 1 for 24 hours, and viral titers were determined by plaque assay (Fig 3A). We observed a significant decrease in KUNV replication in doxycycline-treated Vero *i*-CMPK2 cells compared to mock-treated cells (Fig 3C). We next tested whether the YFV vaccine strain (YFV17D) replication is affected by CMPK2. We observed a significant decrease in YFV17D titers in CMPK2-expressing cells compared to mock-treated Vero *i*-CMPK2 cells (Fig 3D). Thus, CMPK2 inhibits the replication of multiple medically relevant, pathogenic flaviviruses.

Given the broad antiviral activity of CMPK2 against flaviviruses, we asked whether it affects the replication of other virus families that impact global health, including *Coronaviridae*. Neither of the coronaviruses, avian infectious bronchitis virus (IBV) or severe acute respiratory syndrome coronavirus 2 (SARS-CoV-2), the etiological agent of the COVID-19 pandemic [37,38], were inhibited by CMPK2. There were no significant reductions in viral titers of IBV, SARS-CoV-2, and a recombinant SARS-CoV-2 expressing mNeonGreen (SARS-CoV-2-mNG) [39] in CMPK2-expressing cells, measured by plaque assay or by flow cytometry for SARS-CoV-2-mNG (Fig 3A, 3E and 3F). We also asked whether single-stranded, negative-sense RNA viruses are affected by CMPK2 expression. Using GFP reporter viruses of influenza A virus (IAV), a member of the *Orthomyxoviridae* family that causes respiratory diseases, Newcastle disease virus (NDV), a highly contagious avian disease agent belonging to the *Paramyxoviridae* family, and vesicular stomatitis virus (VSV), which belongs to the *Rhabdoviridae* family [40–43], we found no significant difference in the percentage of GFP-positive cells upon CMPK2 induction, as determined by flow cytometry (Fig 3A and 3G–3I). Finally, we examined the effect of CMPK2 on a double-stranded human DNA virus, herpes simplex virus type 1 (HSV-1), belonging to the *Herpesviridae* family that causes a range of clinical symptoms from common cold-sores and fever blisters to encephalitis and neonatal disease involving multiple organs [44]. We found no significant difference in the percentage of GFP-positive cells in CMPK2-expressing cells infected with HSV-1-GFP (Fig 3J) [45]. These results indicate that flaviviruses may be particularly susceptible to CMPK2 antiviral activity. Since infections with flaviviruses are known to trigger oxidative stress and hence affect cellular metabolism and viral replication [46–49], we examined whether CMPK2 expression is linked to elevated mitochondrial reactive oxygen species (mtROS) formation. However, we observed no significant difference in mtROS production measured by MitoSox, a specific dye indicator for mtROS formation in mock or doxy-treated Vero *i*-EV and *i*-CMPK2 cells (S5 Fig). Thus, mtROS production may not be relevant for CMPK2 antiviral activity, at least in our CMPK2 doxycycline-inducible cells. These results indicate that CMPK2 predominantly targets flaviviruses, making it an attractive avenue for the development of broad-spectrum antivirals for flaviviruses.

### Mitochondrial localization of CMPK2 is required for its antiviral function

**Fig 4:**
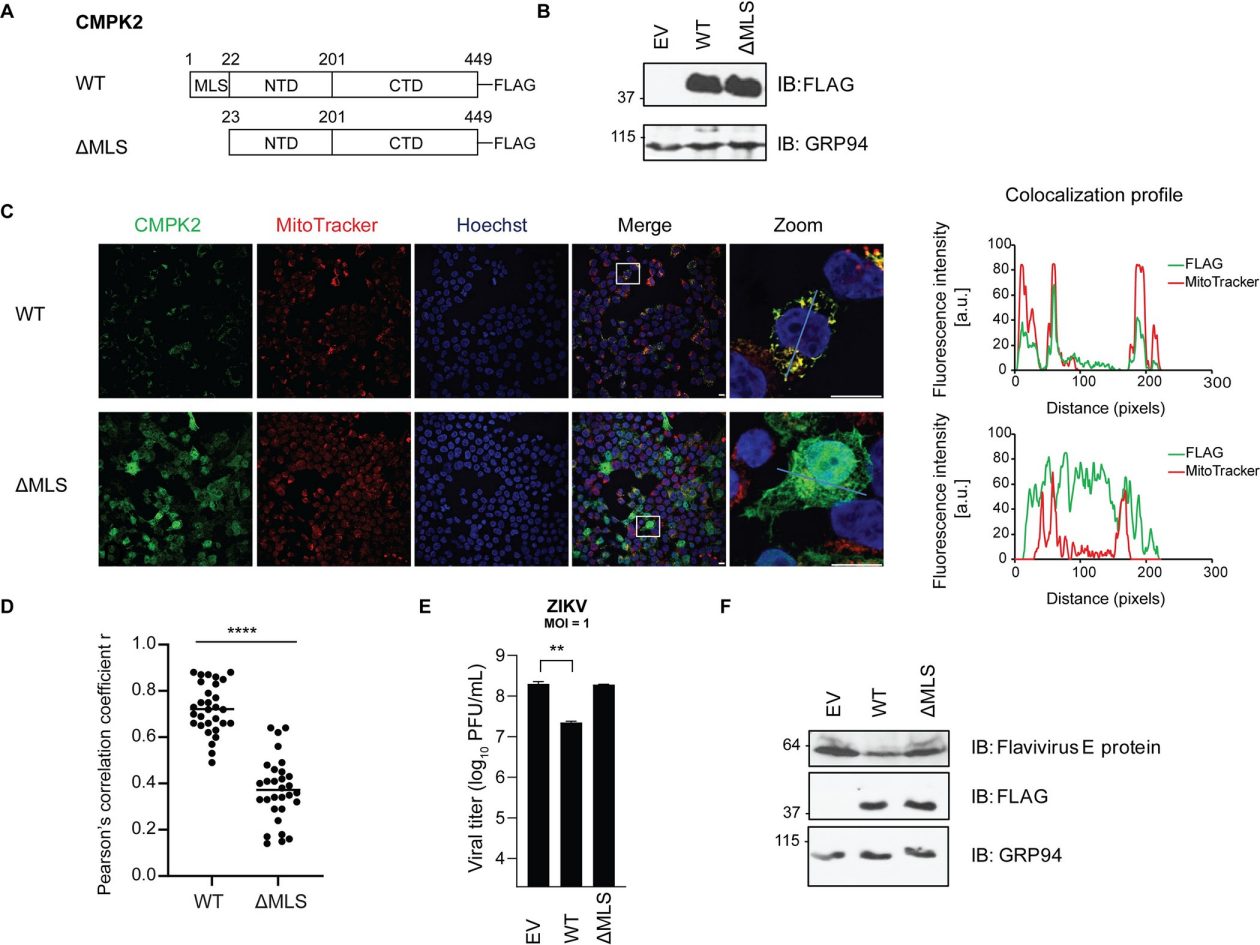
Mitochondrial localization of CMPK2 is required for its antiviral function. **(A)** Schematic illustration of wild-type CMPK2 (CMPK2 WT) and a CMPK2 variant lacking the MLS (amino acid residues 1–22) at the N-terminus (ΔMLS) expression constructs, both bearing a C-terminal FLAG tag. MLS = mitochondrial localization signal. NTD = N-terminal domain; CTD = C-terminal domain. **(B)** Immunoblot analysis of CMPK2 expression in 293T cells transfected with empty vector (EV) control, CMPK2 WT and CMPK2 ΔMLS. At 24 h post transfection, cells were lysed followed by immunoblotting with anti-FLAG for FLAG-tagged CMPK2 and anti-GRP94 antibodies. **(C)** Immunofluorescence analysis of 293T cells transfected with CMPK2 WT and CMPK2 ΔMLS. At 24 h post transfection, cells were treated with MitoTracker (a mitochondrial marker), followed by fixation, permeabilization, and intracellular staining with anti-FLAG antibody. Colocalization of FLAG with MitoTracker is shown in rectangles (zoom) and colocalization profiles are on the right. All images were processed identically using the same settings and colocalization (RGB) profiles were obtained using ImageJ (NIH). a.u. = arbitrary unit. **(D)** Pearson’s correlation coefficient r for FLAG and MitoTracker fluorescence determined in 293T cells transfected with CMPK2 WT and CMPK2 ΔMLS from panel C. Black horizontal bars represent the mean of the group (n = 30). ****p < 0.0001 by unpaired Student’s t test. **(E)** Quantification of Zika virus (ZIKV) production in 293T cells transfected with EV control, CMPK2 WT and CMPK2 ΔMLS. 24 h post transfection, cells were infected with ZIKV at an MOI of 1 for 24 h, and viral titers were determined by plaque assay. Data are shown as mean ± SD of three biological repeats (n = 3). **p < 0.01 by unpaired Student’s t test. MOI = multiplicity of infection; PFU = plaque forming units. **(F)** Immunoblot analysis of ZIKV envelope (E) protein expression in 293T cells transfected with EV control, CMPK2 WT and CMPK2 ΔMLS. 24 h post transfection, cells were infected with ZIKV at an MOI of 1. At 24 hpi, cells were lysed followed by immunoblotting with anti-FLAG, anti-E protein and anti-GRP94 antibodies. hpi = hours post infection.

CMPK2 contains an MLS at the N-terminus (amino acid residues 1–22) [23]. To determine whether its mitochondrial localization is required for CMPK2 antiviral function, a truncated CMPK2 MLS deletion mutant (ΔMLS) was generated (Fig 4A) [23]. 293T cells were transfected with an empty vector (EV) or plasmids expressing WT and ΔMLS CMPK2, all bearing a C-terminal FLAG tag, and subsequently examined by immunoblotting for protein expression (Fig 4B) and confocal immunofluorescence analysis for subcellular localization (Fig 4C). We observed that the expression level of ΔMLS was comparable with that of WT CMPK2 (Fig 4B) and that, as expected, ΔMLS failed to localize in the mitochondria (Fig 4C and 4D). To determine antiviral activities, 293T cells transfected with the EV or expressing WT or ΔMLS CMPK2 were infected with ZIKV at an MOI of 1. Consistent with our results in Vero *i*-CMPK2 cells, WT CMPK2 significantly reduced ZIKV replication compared to cells transfected with an EV (Fig 4E). However, ΔMLS expressing cells had no antiviral activity relative to the EV-transfected cells. Moreover, consistent with the result in Fig 2C, the ZIKV E protein expression was reduced in the cells expressing WT CMPK2 relative to the control and ΔMLS-expressing cells (Fig 4F). Thus, mitochondrial localization and likely a mitochondrial function of CMPK2 is linked to its antiviral activity.

### The N-terminal domain is sufficient for CMPK2 antiviral function

The 22-amino-acid long MLS at the N-terminus of CMPK2 is followed by an N-terminal domain (NTD, amino acid residues 23–201) and a highly conserved C-terminal domain (CTD, amino acid residues 202–449) (Figs S1 and 5A) [23]. The CTD is responsible for CMPK2’s kinase activity [18,23,24]. Replacement of the highly conserved aspartate (D) residue at position 330 in CMPK2’s catalytic pocket with alanine (A) (D330A) results in a catalytically inactive enzyme (S1 Fig) [24].

**Fig 5:**
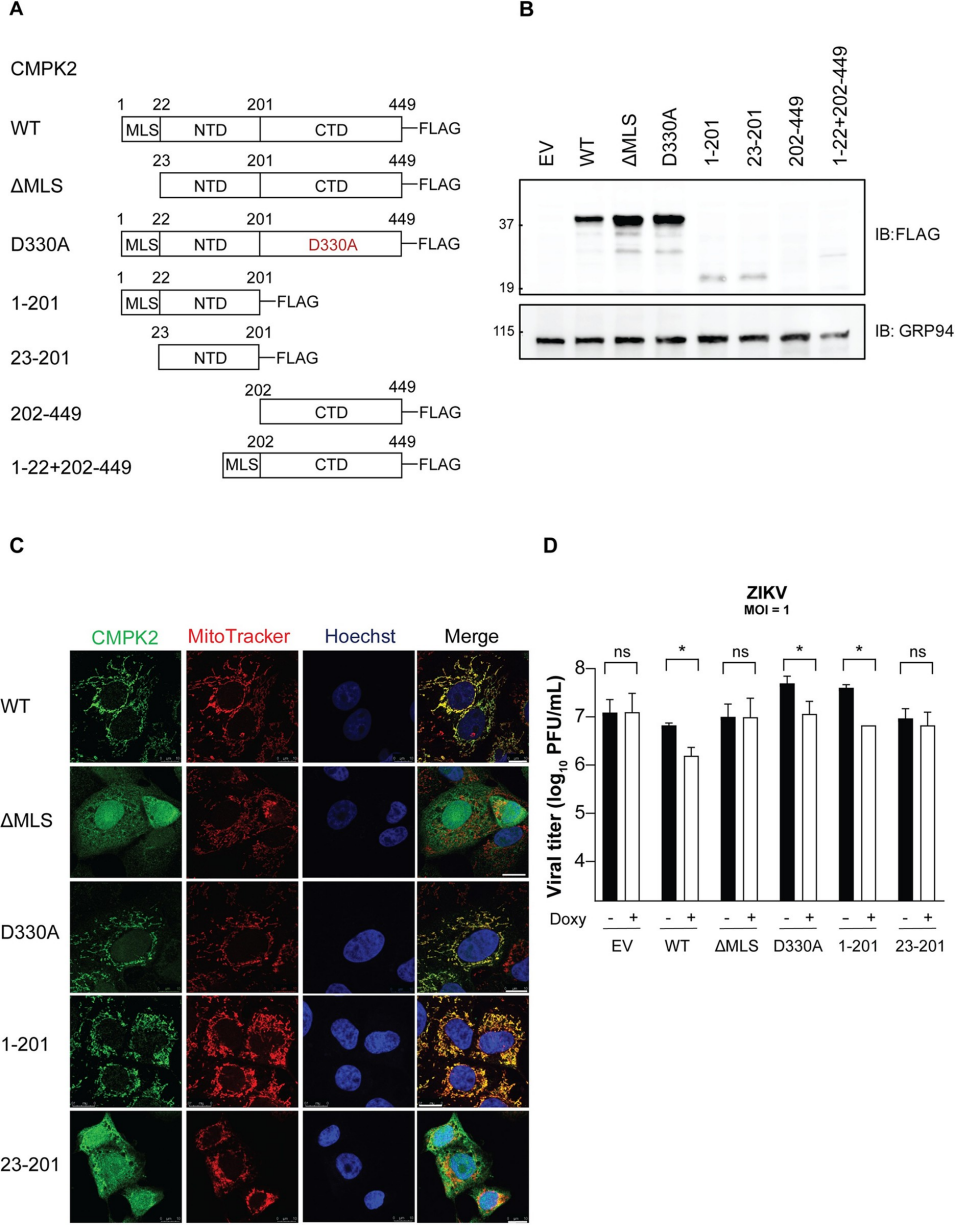
The N-terminal domain is sufficient for CMPK2 antiviral function. **(A)** Schematic illustration of CMPK2 variants used in Fig 5. Wild-type CMPK2 (WT), CMPK2 variant lacking the MLS (ΔMLS), catalytically inactive CMPK2 variant that lacks the kinase activity (D330A), an N-terminal CMPK2 variant (1–201) lacking the CTD; an N-terminal CMPK2 variant (23–201) lacking the MLS and CTD; a C-terminal CMPK2 variant (202–449) lacking the NTD; and a C-terminal CMPK2 variant (1–22+202–449) lacking the NTD but containing the MLS, all bearing a C-terminal FLAG tag. NTD = N-terminal domain; CTD = C-terminal domain. **(B)** Immunoblot analysis of CMPK2 expression in Vero *i-*CMPK2 variant cells that were doxycycline-treated for 24 h. Cell lysates were analyzed by immunoblotting with anti-FLAG and anti-GRP94 antibodies. **(C)** Immunofluorescence analysis of Vero *i-*CMPK2 variant cells that were doxycycline-treated for 24 h then stained with MitoTracker, followed by fixation, permeabilization and intracellular staining with anti-FLAG antibody. **(D)** Quantification of Zika virus (ZIKV) production in Vero *i*-CMPK2 variant cells that were mock- or doxycycline-treated for 24 h then infected with ZIKV at an MOI of 1 for 24 h, and viral titers were determined by plaque assay. Data are shown as mean ± SD of three biological repeats (n = 3). ns = not significant; *p < 0.05 by unpaired Student’s t test. MOI = multiplicity of infection; PFU = plaque forming units; Doxy = doxycycline.

Although the mitochondrial localization of CMPK2 is required for its antiviral activity, it is unknown which domain(s) is responsible for its antiviral function. Therefore, we generated Vero *i*-CMPK2 cell lines expressing CMPK2 truncation mutants to answer this question and to determine whether the kinase activity is essential (Fig 5A). The truncation variants were designed based on the sequence properties as well as the human CMPK2 structure predicted using the AlphaFold protein structure database (Figs 1A and S1) [50]. We generated inducible Vero E6 cell lines expressing CMPK2 mutants lacking the MLS (ΔMLS); lacking the CTD (1–201); lacking the MLS and the CTD (23–201); lacking the NTD (202–449); and lacking the NTD but containing the MLS at the CTD amino terminus (1–22+202–449); all bearing a C-terminal FLAG tag (schematized in Fig 5A). We also generated inducible Vero E6 cells expressing the kinase inactive mutant (D330A) (Fig 5A). The expression of the CMPK2 variants upon doxycycline addition was examined by immunoblot analysis. While we did not attempt to generate a single clonal cell line in order to avoid irreproducible effects, nevertheless, we found differences in construct expression levels. The expression levels of CMPK2 WT, ΔMLS, and D330A were comparable, the expression of 1–201 and 23–201 was less pronounced but comparable to each other, and that 202–449 and 1–22+202–449 were very poorly expressed (Fig 5B). We therefore excluded the latter two mutants from the functional analysis. We also examined the subcellular localization of the variants by confocal microscopy. As anticipated, all mutants that possess the MLS (CMPK2 WT, D330A, 1–201) were localized in the mitochondria, whereas ΔMLS and 23–201 were cytosolic (Fig 5C).

CMPK2 catalyzes the phosphorylation of CDP yielding CTP that is utilized by viperin to synthesize ddhCTP, which acts as a premature chain terminator and inhibits the RNA-dependent RNA polymerase of flaviviruses [18,19]. Moreover, ddhCTP was shown to inhibit ZIKV replication [18]. Although viperin is not expressed in the Vero *i-*CMPK2 experiments described above, we nevertheless asked whether the kinase function of CMPK2 was responsible for its antiviral activity. First, we measured the cellular CTP concentration in doxycycline-treated Vero *i*-CMPK2 variant cells using high-performance liquid chromatography (HPLC). We observed that the CTP concentration, as expected, was significantly increased only in Vero *i*-CMPK2 WT cells (S6 Fig). The CTP concentration in cells expressing the CMPK2 variants lacking the enzymatically active CTD and in the kinase mutant (D330A) was comparable to the concentration in Vero *i*-EV cells (S6 Fig). ΔMLS expressing cells did not produce significant CTP levels despite the presence of CTD, suggesting that the mitochondrial localization may be required for CTP synthesis (S6 Fig). To determine whether the kinase function is crucial for antiviral activity and to establish which CMPK2 domain(s) is responsible for its antiviral activity, Vero *i*-CMPK2 WT and mutant cells were mock- or doxycycline-treated and subsequently infected with ZIKV at an MOI of 1 for 24 h. Given that the CMPK2 variant cell lines differ in CMPK2 expression levels, it may be that the cell infectivity is at variance too. Hence, to keep the results as stringent as possible, we compared the ZIKV titers and performed statistical analysis of mock-treated versus doxy-treated cells within the same CMPK2 construct cell line. Consistently, cells expressing WT CMPK2 inhibited ZIKV replication, whereas ΔMLS failed to do so (Fig 5D). Notably, the kinase inactive D330A mutant also restricted ZIKV replication (Fig 5D), suggesting that CTP generation is not required for the antiviral activity of CMPK2. Moreover, the 1–201 variant, which is primarily localized in the mitochondria but lacks the C-terminal kinase domain, restricted ZIKV replication but the 23–201 variant encoding the N-terminal domain but lacking the MLS did not (Fig 5D). These results show that the N-terminal domain of CMPK2 is sufficient for its antiviral function but only when targeted to the mitochondria.

### Cysteine residues within the NTD are required for CMPK2 antiviral activity

**Fig 6:**
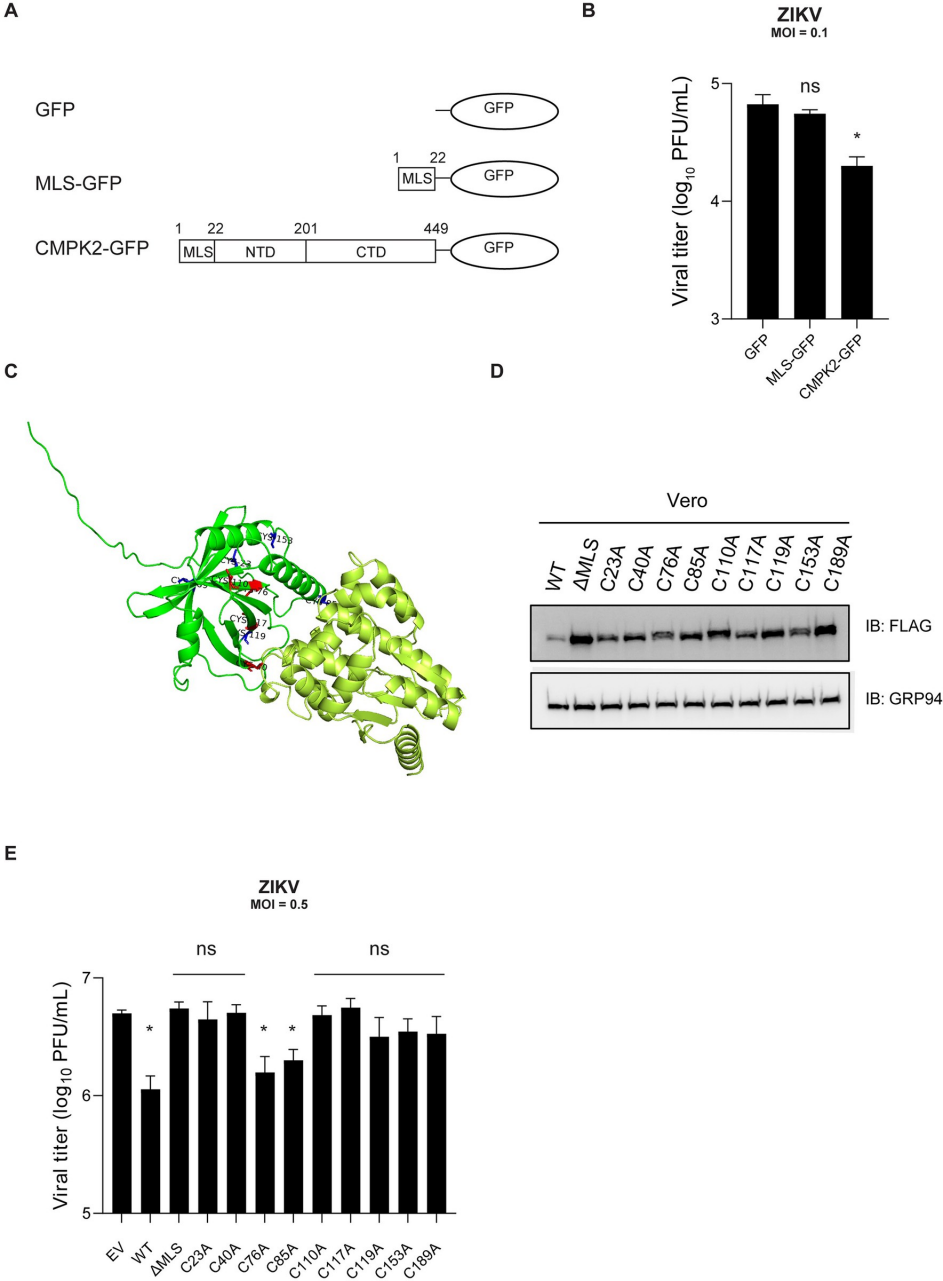
Cysteine residues within the NTD are required for CMPK2 antiviral activity. **(A)** Schematic illustration of CMPK2/GFP variants; GFP-plasmid, MLS-GFP (first 22 amino acid of CMPK2 protein conjugated to GFP) and CMPK2-GFP used in Fig 6B. **(B)** Quantification of ZIKV production in Vero cells expressing GFP-only, GFP-conjugated MLS and CMPK2 constructs. Vero cells were transfected with the indicated plasmids, sorted for GFP-expressing cells, and infected with ZIKV at an MOI of 0.5. Viral titers were determined by plaque assay at 24 hpi. hpi = hours post infection; MOI = multiplicity of infection; PFU = plaque forming units. **(C)** AlphaFold predicted human CMPK2 structure with indicated in red buried and in blue exposed 9 cysteine residues. **(D)** Immunoblot analysis of CMPK2 NTD cysteine to alanine mutants. Vero cells were transfected with the indicated plasmids followed by immunoblot analysis using the indicated antibodies. **(E)** Quantification of ZIKV production in Vero cells expressing CMPK2 NTD cysteine to alanine variants. Vero cells were transfected with the indicated plasmids, then infected with ZIKV at an MOI of 0.5 and viral titers were determined by plaque assay. Data are shown as mean ± SD of three biological repeats (n = 3). ns = not significant; *p < 0.05 by unpaired Student’s t test. MOI = multiplicity of infection; PFU = plaque forming units.

To evaluate whether the MLS contributes to CMPK2 antiviral function against ZIKV, we generated a GFP-plasmid, GFP-conjugated MLS (first 22 amino acid residues of CMPK2 protein) and CMPK2-GFP plasmid (Fig 6A). Vero cells were transfected with these plasmids, sorted for GFP-expressing cells, and infected with ZIKV at an MOI of 0.1. Plaque assay was used to determine ZIKV titers at 24 hpi. The results show that CMPK2-GFP significantly reduces ZIKV replication compare to the GFP-only control (Fig 6B). Moreover, we observed no significant difference between GFP-conjugated MLS and GFP-only control (Fig 6B). These results indicate that expression of only the MLS of CMPK2 does not affect ZIKV replication but rather the MLS is required to transport at least the NTD of CMPK2 into the mitochondria where CMPK2 can execute its antiviral function.

Notably there are nine cysteine residues located in the first 189 amino acids residues of the NTD (Fig 6C). To determine whether the NTD cysteine residues are required for CMPK2 antiviral activity, we made single cysteine to alanine (Cys-Ala) mutants of all nine cysteines. We first verified their expression by immunoblot analysis (Fig 6D). Vero cells were then transfected with the EV, WT, ΔMLS and Cys-Ala CMPK2 mutants and infected with ZIKV at an MOI of 0.5. Viral titers were measured at 24 h post infection by plaque assay. We identified two (C76 and C85) out of nine cysteines that are not required for CMPK2 antiviral activity against ZIKV (Fig 6E). The Cys-Ala mutations of the remaining seven cysteine residues abolished CMPK2 antiviral function, implying that those residues are essential for CMPK2 to inhibit ZIKV.

### CMPK2 inhibits ZIKV translation

**Fig 7:**
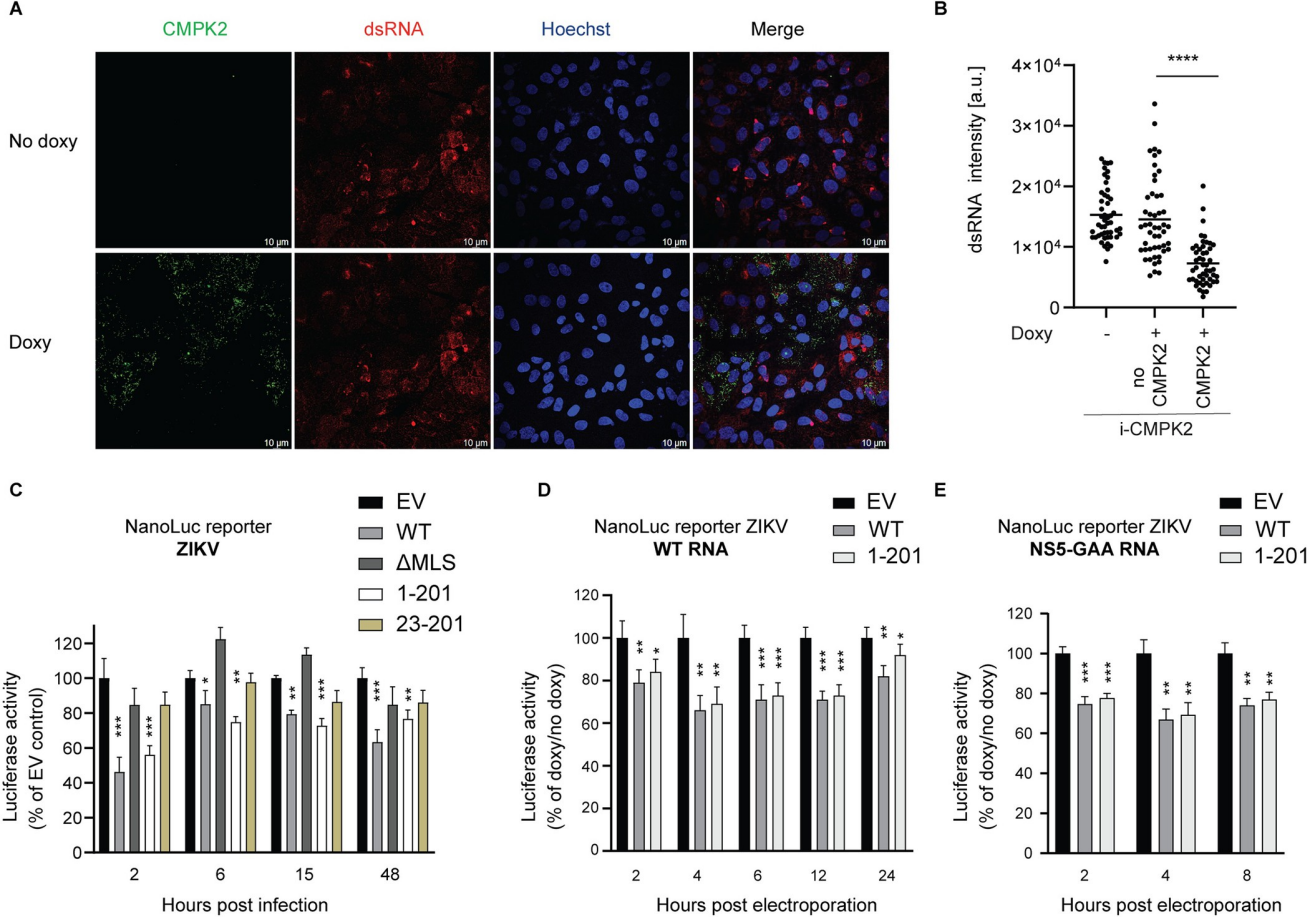
CMPK2 inhibits Zika virus translation. **(A)** Immunofluorescence analysis of Vero *i-*CMPK2 cells that were mock- or doxycycline-treated for 24 h then infected with ZIKV at an MOI of 1. 48 hpi cells were fixed, permeabilized and stained by anti-FLAG antibody for CMPK2 and J2 antibody for dsRNA detection. MOI = multiplicity of infection; hpi = hours post infection; Doxy = doxycycline. Uninfected control in S7A Fig. **(B)** Quantification of the dsRNA fluorescence intensities in Vero *i*-CMPK2 cells from panel A. Total dsRNA intensity is quantified as (cell area* mean dsRNA intensity). Black horizontal bars represent the mean of the group (n = 49). ****p < 0.0001 by unpaired Student’s t test. No CMPK2 = doxycycline-treated but no CMPK2 expression present. a.u. = arbitrary unit. **(C)** Luciferase activity assay in Vero *i*-CMPK2 variant cells that were doxycycline-treated for 24 h then infected with NanoLuc reporter ZIKV at an MOI of 0.5 for 1 h. At indicated time post infection, the cells were lysed and measured for luciferase activities. Data are shown as mean ± SD of three biological repeats (n = 3). *p < 0.05, **p < 0.01, ***p < 0.001 by unpaired Student’s t test. **(D)** Luciferase activity assay in Vero *i*-EV, -CMPK2 and 1–201 variant cells that were doxycycline-treated for 24 h then electroporated with the *in vitro* transcribed RNA encoding a luciferase reporter ZIKV (NanoLuc reporter ZIKV RNA). At indicated time post electroporation, the cells were lysed and measured for luciferase activities. Data are shown as mean ± SD of three biological repeats (n = 3). *p < 0.05, **p < 0.01, ***p < 0.001 by unpaired Student’s t test. **(E)** Luciferase activity assay in Vero *i*-EV, -CMPK2 and 1–201 variant cells that were doxycycline-treated for 24 h then electroporated with the *in vitro* transcribed ZIKV NS5 GAA mutant RNA encoding a luciferase reporter ZIKV (NanoLuc reporter ZIKV NS5 GAA RNA). At indicated time post electroporation, the cells were lysed and measured for luciferase activities. Data are shown as mean ± SD of three biological repeats (n = 3). **p < 0.01, ***p < 0.001 by unpaired Student’s t test.

Our results showed that upon ZIKV infection, cells expressing CMPK2 have reduced E protein expression and significantly lower viral titers as compared to control. We obtained similar results by assessing ZIKV double-stranded RNA (dsRNA) formed during viral RNA replication by immunofluorescence imaging using J2 antibody. Vero *i*-CMPK2 cells that were doxycycline-treated and expressed CMPK2 had significantly less ZIKV dsRNA present at 48 hpi as compared to non-CMPK2 expressing cells (Figs 7A and 7B and S7A). Taken together, CMPK2 expressing cells had reduced ZIKV RNA levels. However, it was not apparent which step in the ZIKV life cycle prior to RNA synthesis was affected. To test this, we employed a NanoLuc luciferase reporter ZIKV system to distinguish between viral RNA translation and RNA replication [51]. The NanoLuc reporter ZIKV system was chosen due to its superior sensitivity; its activity can be detected as early as 2 hpi. The NanoLuc signals at 2–6 hpi are an estimation of the level of viral RNA translation, while NanoLuc activity after 12 h assesses the level of viral RNA replication [51,52]. We treated the Vero *i*-CMPK2 WT and mutant cells with doxycycline and subsequently infected them with the NanoLuc reporter ZIKV at an MOI of 0.5 for 1 h. First, we verified whether there is any significant difference in cell viability in doxycycline-treated Vero *i*-EV and *i*-CMPK2 variant cells upon NanoLuc luciferase reporter ZIKV infection. We used a luminescent cell viability assay that quantifies ATP present in culture to determine the number of viable cells. We observed no significant change in ATP production in doxycycline-treated Vero *i*-EV and *i*-CMPK2 variant cells upon NanoLuc luciferase reporter ZIKV infection (S7B Fig), suggesting that CMPK2-expressing cells are viable. It was revealed that CMPK2 WT and the 1–201 variant significantly inhibit the NanoLuc signal as early as 2 hpi, which represents/reflects the initial translation of ZIKV RNA (2 and 6 hpi), and also at 48 hpi, when viral replication is completed (Fig 7C). This result indicated that CMPK2 inhibits ZIKV infection early in the viral life cycle, likely viral translation. However, we could not rule out the possibility that CMPK2 inhibits other early infection stages such as virus entry or membrane fusion. To bypass those steps we electroporated the mock- and doxycycline-treated Vero *i*-EV, -CMPK2 and the 1–201 variant cells with *in vitro*-transcribed ZIKV RNA encoding a luciferase reporter (NanoLuc reporter ZIKV RNA). We observed that the NanoLuc activity was inhibited in doxycycline-treated Vero *i*-CMPK2 and 1–201 cells compared to doxycycline-treated Vero *i*-EV cells as early as 2 h and up to 24 h post electroporation (Fig 7D). This result suggested that CMPK2 inhibits ZIKV RNA translation. In addition, we used NanoLuc reporter ZIKV RNA containing the nonstructural protein 5 (NS5) conserved polymerase active site G664DD residues mutated to G664AA resulting in a replication-incompetent mutant (NS5-GAA) as a control for CMPK2 direct effect on viral translation [51,52]. The electroporation of NS5-GAA replication-incompetent mutant RNA also resulted in inhibition of NanoLuc activity in doxycycline-treated Vero *i*-CMPK2 and 1–201 cells compared to doxycycline-treated Vero *i*-EV cells as early as 2 h post electroporation (Fig 7E). The NanoLuc signal began to decrease after approximately 8 h post electroporation due to lack of replication (S7C Fig) [53]. Taken together, these results support our conclusion that CMPK2 inhibits viral translation. To further complement those findings, we examined whether CMPK2 affects events in ZIKV life cycle prior to translation. A virus entry assay was performed as reported by Le Sommer *et al*. [54]. Vero *i*-EV and *i*-CMPK2 cells were doxycycline-treated and subsequently infected with ZIKV at an MOI of 5 for 2 h at 37°C. Unbound/uninternalized virus particles were removed and total RNA was extracted and analyzed by qRT-PCR. We found no significant difference in ZIKV RNA levels between doxycycline-treated cells (S7D Fig.). In addition, we checked whether CMPK2 expression affects endosome-virus membrane fusion using a lipophilic dye (DiOC18) to label ZIKV [55]. Vero *i*-EV and *i*-CMPK2 cells were mock- and doxycycline-treated and subsequently infected with DiOC18-labelled ZIKV at an MOI of 1 for 1 h at 37°C. As expected, CMPK2 expression did not significantly attenuate endosome-virus membrane fusion (S7E Fig). Taken together, these viral life cycle studies revealed that CMPK2 inhibits ZIKV RNA translation.

A recent study showed that viperin, through its radical S-adenosyl methionine (SAM) activity, inhibits both viral and cellular RNA translation to restrict flavivirus replication [17]. Given the functional linkage between CMPK2 and viperin, we asked whether CMPK2 also inhibits cellular translation. To test this we measured the expression of Firefly luciferase (Fluc) in Vero *i*-EV and *i*-CMPK2 cells transfected with *in vitro* transcribed Fluc reporter mRNA. We found no significant difference in luciferase activity between doxycycline-treated cells 6 h and 24 h post transfection (S8A Fig), suggesting that CMPK2 does not inhibit cellular translation. We confirmed this at the single cell level using O-propargyl-puromycin (OP-Puro) click chemistry labeling [12,17,56–58] that measures global translation activity of endogenous mRNA. We showed by flow cytometry that 293T cells expressing CMPK2 had comparable translation activity (protein synthesis levels) to the cells expressing EV (S8B Fig). These results suggest that CMPK2 inhibits ZIKV life cycle by predominantly targeting viral translation rather than host translation.

## Discussion

CMPK2 is an antiviral ISG-encoded protein. It was previously reported that cells transfected with small interfering RNAs (siRNAs) to CMPK2 exhibited suppressed IFN-induced restriction of HIV [27]. In addition, Lai *et al*. showed that infected CMPK2-KO cells had elevated expression of DENV RNA and that CMPK2 restricts DENV replication [21]. However, no mechanism of action for CMPK2 antiviral activity was elucidated in those studies. We focus here on the scope of CMPK2’s antiviral activities and the functional characterization of CMPK2 in the context of flavivirus infection. We showed that CMPK2 restricts an early step in the life cycle of ZIKV, specifically, ZIKV RNA translation and that CMPK2 is required to inhibit ZIKV during the IFN-I response. Furthermore, we demonstrated that CMPK2 inhibits other pathogenic flaviviruses including DENV-2 which is consistent with the literature, KUNV and YFV, suggesting that flaviviruses in general may be susceptible to the CMPK2 antiviral function. Influenza A virus, vesicular stomatitis virus, Newcastle Disease virus, two different coronaviruses (SARS-CoV-2 and IBV) and herpes simplex virus type I were unaffected by CMPK2 expression suggesting specificity to flaviviruses and not a general antiviral effect. Li *et al*. reported that chicken CMPK2 inhibits avian influenza and NDV in chicken fibroblasts [29]. However, in our experimental system using human CMPK2 in African green monkey fibroblasts we did not observe this effect. This may be due to cell type differences as well as differences in the CMPK2 protein between species. Full length chicken CMPK2 and human CMPK2 are 60% identical at the amino acid level. In addition, chicken CMPK2 has a partial NTD of about 70 amino acids and has only two of the nine cysteine residues of human CMPK2 corresponding to C153 and C189 [59]. Therefore, it is possible that chicken CMPK2 may affect IAV and NDV in chicken cells via the distinct NTD. Of note, we did not examine the effect of CMPK2 on HIV replication in our studies however CMPK2 may also affect HIV viral translation or it may affect a step in viral genome amplification. What confers the specificity of CMPK2-mediated inhibition of flavivirus replication is unknown. Whether hepatitis C virus which belongs to the *Flaviviridae* family will also be affected by CMPK2 remains to be examined.

Our work revealed a novel antiviral function of CMPK2 where the N-terminal domain, independently of the kinase domain, is sufficient to inhibit ZIKV translation when localized to the mitochondria. Of note, the expression level of CMPK2 variants upon induction is not uniform. However, increased ISG expression levels do not necessarily correlate with improved functional antiviral activities [60]. The 1–201 and WT CMPK2 exhibited similar antiviral activity despite different degree of expression whereas 23–201 has comparable expression to 1–201 but failed to inhibit ZIKV replication. Thus, it is unlikely that decreased expression of 23–201 is responsible for the absence of its antiviral activity. It may be that CMPK2 antiviral activity has already reached its maximum efficacy and higher levels of CMPK2 expression may not necessarily enhanced its antiviral activity. In addition, we identified seven conserved cysteine residues within the NTD that are critical for CMPK2 antiviral activity indicating a unique function within the NTD that likely contributes to CMPK2 antiviral activity.

CMPK2 is the product of a nuclear gene that contains an N-terminal MLS. We determined that the mitochondrial localization of CMPK2 is required for its antiviral activity against ZIKV (Figs 4 and 5) however expression of only the MLS have no effect on ZIKV replication. While it is conceivable that CMPK2 expressed in the cytosol is simply misfolded and non-functional, it seems likely that mitochondrial localization is essential for its antiviral effects. However, *in vitro* studies involving recombinant CMPK2 revealed that CMPK2 phosphorylates CDP and UDP, yielding CTP and UTP respectively, suggesting that CMPK2 lacking the MLS (non-mitochondrial CMPK2) can be functional and active [18].

Flavivirus infections disturb mitochondrial dynamics and trigger oxidative stress, which affects cellular metabolism and can activate apoptosis and autophagy pathways [61,62]. For example, ZIKV infections induce mitochondrial fragmentation and an imbalance in fusion/fission equilibrium in human retinal cells [61]. DENV nonstructural protein NS4B associates with mitochondrial proteins causing mitochondrial elongation to promote DENV replication [63]. Another ISG, ISG12b2, which is localized to the inner mitochondrial membrane was recently reported to exhibit antiviral effects against DENV. Overexpression of mouse ISG12b2 or induction of ISG12b2 upon DENV infection led to the initiation of mitochondria-mediated apoptosis [64]. CMPK2 expression in our doxycycline-inducible cells seems to have no effect on ATP production and thus cell viability, suggesting that CMPK2-expressing cells are metabolically active (S7B Fig). Finally, infections with DENV have been found to induce the production of mtROS leading to inflammasome activation in THP-1 cells which is suppressed in THP-1 CMPK2-KO cells [21]. The inflammasome is crucial to host defense against viral pathogens as it promotes IL-1β secretion, which leads to recruitment of neutrophils and elimination of virally-infected cells [65]. The production of mtROS and subsequent release of mtDNA into the cytoplasm activates several different pattern recognition receptors and innate immune responses, including the IFN-I response [66]. For example, infections with HSV-1, VSV and DENV-2 result in mtDNA release into the cytoplasm, which is then sensed by cGAS, subsequently leading to upregulation of ISGs [66,67]. The lack of an effect of CMPK2 expression on HSV-1 and VSV infections observed here (Fig 3) suggests that cytosolic release of mtDNA is unlikely to be relevant to its viral inhibitory activity. Nevertheless we checked whether the expression of CMPK2 causes elevated mtROS formation. As expected, no significant difference in mtROS production was observed upon CMPK2 expression (S5 Fig), suggesting that mtROS production may not be relevant for CMPK2 antiviral activity. Nonetheless, CMPK2 may have another effect(s) on mitochondrial function that may be important. Understanding the effects of CMPK2 on cellular metabolism, including mitochondrial function and the cellular metabolome may lead to a larger perspective of CMPK2 mechanism of action.

There is a functional linkage between CMPK2 and viperin however whether CMPK2 can establish an antiviral state, independent of viperin, is unclear. CMPK2 phosphorylates CDP to yield CTP, which can be used as a substrate by viperin to produce 3’-deoxy-3’,4’-didehydro-cytidine triphosphate (ddhCTP) that acts as a chain terminator for the RNA-dependent RNA polymerases of multiple flaviviruses [18]. Our experiments involving Vero *i*-CMPK2 cells, where CMPK2 but no viperin is expressed upon doxycycline treatment, indicated that CMPK2 exhibits its antiviral effects independently of viperin. Nevertheless, we asked whether its kinase activity is required for its antiviral function and found that the well-conserved C-terminal kinase domain is not essential for its antiviral activity against ZIKV (Fig 5D). We demonstrated that the CMPK2 N-terminal domain, which has no known biological function, mediates antiviral activity against ZIKV but only when it is primarily localized to the mitochondria (Figs 5D and 7C). The N-terminal domain is rich in leucine, alanine, proline and glycine residues [23], which all are prone to form intermolecular hydrophobic interactions [68]. Although no leucine-zipper motif has been identified in the NTD, the proteins with more buried hydrophobic inner space tend to be involved in protein-protein interactions (S1 Fig) [23,69,70]. The NTD has a low degree of sequence homology within the NCBI protein database but has highly conserved cysteine residues across the mammalian genomes that we determined to have a significant role in CMPK2 antiviral response (Figs 6E and S1). Cysteine residues typically indicate the possibility of disulfide bond formation or metal binding capacities [25]. However, based on the AlphaFold prediction, the cysteine residues required for CMPK2 antiviral activity do not form disulfide bonds, indicating an unknown functional site within the NTD that is required for CMPK2 antiviral activity. The existence of two distinctive domains with different characteristics suggests that CMPK2 may be a bifunctional protein with two molecular/biological functions, exerting direct antiviral effects through its N-terminal domain while the kinase function of its C-terminal domain provides CTP for viperin. Further studies are required to elucidate how these two domains coordinate with each other and cellular networks to establish an antiviral state against flavivirus infections. Given that CMPK2 expression and localization vary between cell types and species [21,29,71], CMPK2 may have diverse biological roles depending on the cell type in which it is expressed and the physiological responses it mediates.

We showed that CMPK2 inhibits ZIKV translation (Fig 7). It has been shown that several ISG-encoded proteins inhibit translation [13,17,72]. Translational regulation can be accomplished by suppression of viral or cellular translation or both. For example, to inhibit viral translation, the interferon-induced protein with tetratricopeptide repeats 1 (IFIT1) suppresses translation initiation via direct interactions with eukaryotic initiation factors (eIFs) [73–75]. Certain viruses alter cellular tRNA pool by skewing codon bias toward viral genomes to facilitate viral translation. The ISG-encoded protein schlafen 11 (SLFN11) counteracts this alteration by perhaps binding to these modified tRNAs and thereby inhibiting viral translation [76]. To restrict flavivirus replication, viperin through its enzymatic product ddhCTP triggers ribosome collision, leading to activation of the integrated stress response (ISR), which ultimately suppresses both viral and cellular RNA translation [17]. Some ISG-encoded proteins, such as NEDD4-binding protein 1 (N4BP1) and ZAP, specifically target viral RNA translation by degrading viral RNA [15,77]. Our studies revealed that CMPK2 establishes an antiviral state disfavoring viral RNA translation, which may serve as a potent antiviral defense mechanism against flaviviruses. However, we cannot exclude the possibility that the reduction in viral RNA translation may not be responsible for the entirety of the antiviral phenotype. Further studies that focus on RNA degradation and/or metabolic effects of CMPK2 on the mitochondria will shed more light on the mechanism by which CMPK2 mediates inhibition of flaviviruses. Whether CMPK2 expression in the mitochondria affects a general cellular/subcellular phenomenon that consequently affects flaviviruses downstream of the mitochondria is unknown. It is also possible that expression of CMPK2 leads to the upregulation of RNA binding proteins that specifically bind the flavivirus genome and not the genome of other viruses like SARS-CoV-2.

**Fig 8:**
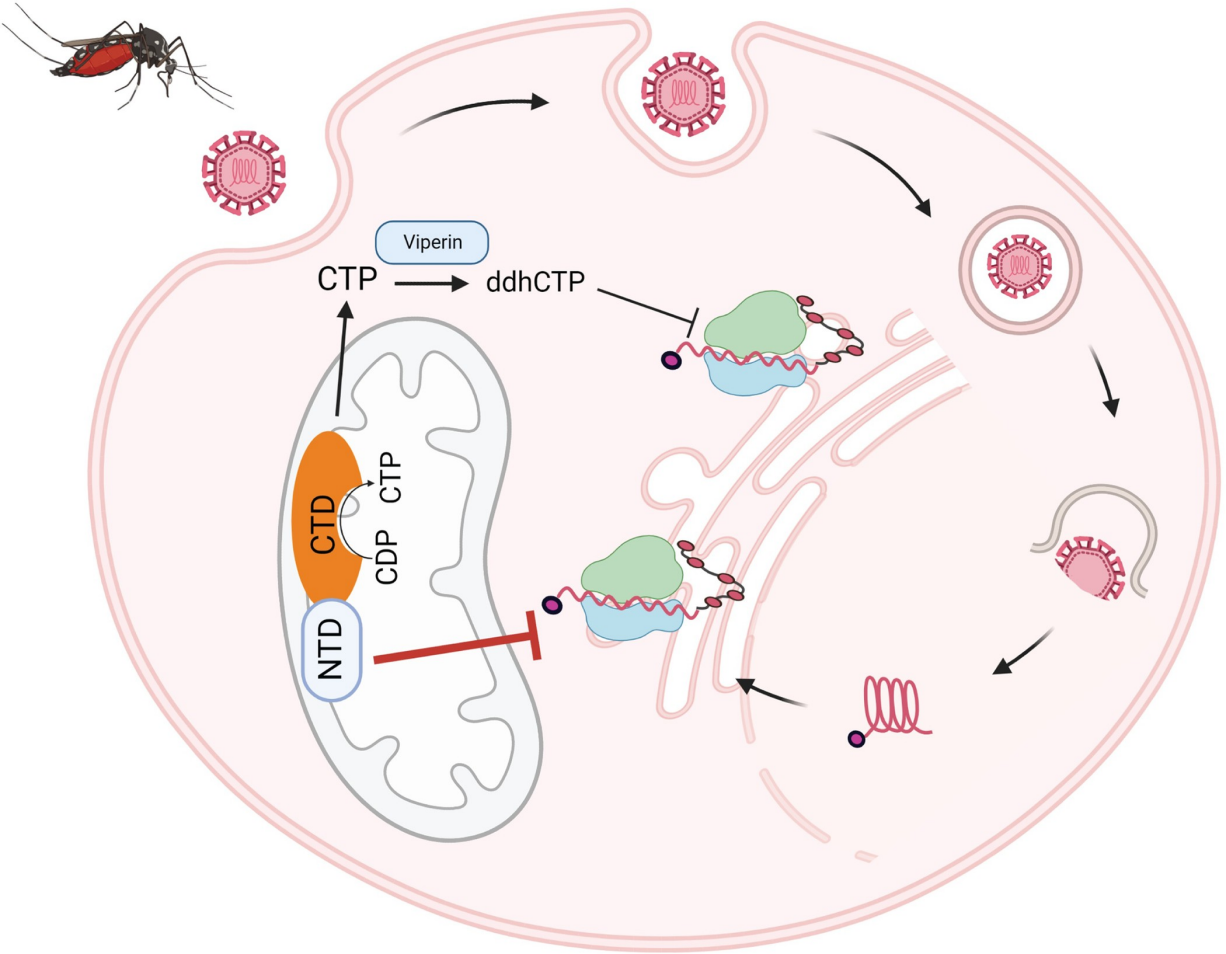
Working model for the effects of mitochondrial CMPK2 on flavivirus infections. Zika virus enters cells by receptor-mediated endocytosis and traffics to endosomes where the acidic environment induces fusion of the viral and host membranes. The viral RNA is released and RNA translation occurs at the surface of the ER. CMPK2 translocates to the mitochondria where it exerts its antiviral activity through both its N-terminal domain (NTD) and C-terminal domain (CTD). The CTD possesses kinase activity and produces cytidine triphosphate (CTP), the substrate for viperin. Viperin converts the CTP to 3’-deoxy-3’,4’-didehydro-cytidine triphosphate (ddhCTP) which inhibits virus and host cell translation [17]. The NTD of CMPK2 is sufficient to restrict Zika virus replication independent of the CTD by specifically inhibiting viral RNA translation. Illustration was created with BioRender.com.

A working model for the effects of CMPK2 on flaviviruses, where a mitochondrial localized N-terminal domain of CMPK2 is sufficient to inhibit flavivirus replication by potential interactions with RNA-binding protein(s) is presented in Fig 8. It is feasible that a CMPK2 interacting partner(s) or a product akin to ddhCTP is released from the mitochondria into the cytosol where it recognizes and binds to specific RNA sequences of flaviviruses thereby inhibiting viral translation. Currently, efforts are being made to identify and characterize host and viral factors that may interact with CMPK2, particularly the N-terminal domain, during viral infection and to elucidate whether such factors regulate its antiviral activity. Dissection of the mechanism by which CMPK2 inhibits flavivirus translation may yield new strategies for adapting the antiviral activity of CMPK2 into clinically useful, broad-spectrum flavivirus therapeutics.

## Materials and methods

### Constructs

pcDNA3.1CMPK2-WT-FLAG and pcDNA3.1CMPK2-ΔMLS-FLAG were purchased from GenScript. To generate doxycycline-inducible cell lines, a pTRIPZ lentivirus was used to transduce Vero E6 cells. For CRISPR-Cas9 knockout, lentiviruses were generated with pCMV-VSV-G (Addgene, catalog no. 8454), psPAX2 (Addgene, catalog no. 12260) and guide RNA encoding plasmids pSpCas9(BB)-2A-GFP vector (Addgene, catalog no. 48138) or non-targeting control gRNA (Addgene, catalog no. 80263). All primers used for molecular cloning and qRT-PCR were synthesized by the Keck facility at Yale University and are listed in S1 Table. Plasmids were purified using Zymo Research kits.

### Antibodies

The primary antibodies used were rabbit anti-hCMPK2 (peptide sequence: amino acids 48–61 made by GenScript), anti-Flavivirus E protein (MAB10216; Millipore), anti-FLAG (F7425; Sigma), anti-viperin (MaP.VIP) [78], anti-RIG-I (3743; Cell Signaling Technology), anti-GRP94 (9G10; Enzo), anti-GAPDH (10494-1-AP; Proteintech) and anti-dsRNA J2 (RNT-SCI-10010200; Jena Bioscience). All secondary antibodies used for immunofluorescence imaging and immunoblotting were purchased from Invitrogen and Jackson ImmunoResearch, respectively.

### Cells

293T, Vero and Vero E6 cells were purchased from ATCC. Human foreskin fibroblast (HFF) cells were previously reported in Bresnahan *et al*. [32]. Cells were grown in Dulbecco’s modified Eagle’s medium (DMEM; Gibco) supplemented with 10% fetal bovine serum (FBS; HyClone). For DNA plasmid transfection, cells were plated one day before and transfected using Lipofectamine 2000 according to the manufacturer’s instructions. For ISG induction, Vero E6 cells were treated with 1,000 U/mL of Universal Type I IFN (PBL) for 18 h. To generate doxycycline-inducible cell lines, a pTRIPZ lentivirus was used to transduce Vero E6 cells. Cells were selected with puromycin (Thermo Fisher Scientific) at 5 μg/mL to obtain stable cell lines. To generate CMPK2-knockout cell lines, HFF cells were transduced with a lentivirus encoding the guide RNA of interest. Cells were selected by sorting for GFP-positive cells.

### Viruses and cell culture infections

Virus stocks used in this study include: Cambodian Zika virus (ZIKV) strain: FSS13025, which was generously provided by Dr. Erol Fikrig at Yale University. WNV-KUNV strain: CH16532 was obtained from Dr. Philip M. Armstrong at the Connecticut Agricultural Experiment Station. Vesicular stomatitis GFP virus (VSV-GFP) and Herpes simplex type 1 GFP virus (HSV-1-GFP) were kind gifts from Dr. Akiko Iwasaki at Yale University. Yellow fever vaccine strain 17D virus (YFV17D), dengue virus serotype 2 (DENV-2) strain 16681, Newcastle disease GFP virus (NDV-GFP) and influenza A virus (IAV) expressing a fluorescent protein (PR8-GFP) were kind gifts from Dr. Adolfo García-Sastre at the Icahn School of Medicine. Avian *infectious bronchitis virus* (IBV) was provided by Dr. Carolyn Machamer at the Johns Hopkins University School of Medicine. High-titer stocks of SARS-CoV-2 virus (isolate USA-WA1/2020, CoV-2 WA1) were obtained from BEI reagent repository and SARS-CoV-2-mNG [39] was generated by passage in Vero E6 cells. Viral titers were determined by plaque assay on Vero E6 cells. Virus was cultured exclusively in a biosafety level 3 facility. High titer stocks of Zika Virus (ZIKV), Cambodia strain were obtained by passage in Vero cells. High titer stocks of KUNV, DENV-2 and YFV17D were obtained by passage in BHK-21 cells. Monolayers of cells were initially adsorbed with virus at the indicated multiplicity of infection (MOI) in 2% FBS/DMEM for 1 h at 37°C. Unbound virus was removed, and cells were maintained in 10% FBS/DMEM at 37°C. Viral replication was measured by plaque assay on Vero cells for ZIKV or BHK-21 cells for WNV_KUNV_, DENV-2 and YFV17D. For doxycycline-inducible Vero E6 cell lines, cells were induced using 2 μg/mL doxycycline (Sigma) for 24 h following the infection with the indicated virus and at the indicated multiplicity of infection (MOI) in 2% FBS/DMEM or serum-free DMEM (for SARS-CoV-2) for 1 h at 37°C. Unbound virus was removed, and cells were maintained in 10% FBS/DMEM or in 2% FBS/DMEM (for SARS-CoV-2) containing 2 μg/mL doxycycline at 37°C for 24 h. Viral replication was measured by plaque assay on Vero E6 cells or BHK-21 cells or by flow cytometry for GFP viruses.

For a luciferase reporter ZIKV (NanoLuc reporter ZIKV) infection [51], cells were infected at an MOI of 0.5 for 1 h at 37°C. The inoculations were removed, cells were washed twice with PBS and maintained in 10% FBS/DMEM containing 2 μg/mL doxycycline. At indicated time post infection, the cells were harvested, lysed and measured for luciferase activities in a luminescence micoplate reader (PerkinElmer) according to the manufacturer’s protocol [79].

For a luciferase reporter ZIKV RNA (NanoLuc reporter ZIKV RNA) studies, cells (1e7) were electroporated with 10 μg of the RNA in Ingenio Electroporation Solution (Mirus, Madison, WI) as described in Shan *et al*. [80]. At indicated time post electroporation, the cells were harvested, lysed and measured for luciferase activities in a luminescence micoplate reader (PerkinElmer) according to the manufacturer’s protocol [79].

### Immunoblotting

Cells were lysed in Laemmli sample buffer and boiled. Cell lysates were separated by SDS-PAGE on 12% homemade gels or 4 to 15% gradient gel (Bio-Rad) and transferred onto PVDF membranes (Millipore). Membranes were blocked in 5% bovine serum albumin (BSA)/Tris-buffered saline with 0.1% Tween 20 (TBST) for 1 h at RT, incubated with primary antibodies in 2% BSA/TBST for overnight at 4°C, incubated with secondary horseradish peroxidase-conjugated antibodies in 2% BSA/TBST for 1 h at RT, and analyzed by a Chemidoc MP Imaging System (Bio-Rad).

### Confocal immunofluorescence microscopy

Cells were grown on glass cover slips and following indicated treatment were washed with PBS and fixed with 4% paraformaldehyde in PBS for 15 min at room temperature followed by three washes with PBS. Cells were permeabilized with 0.5% Triton X-100 in PBS for 15 min at room temperature followed by three washes with PBS and blocked with 5% FBS, 0.05 M glycine in PBST (0.5% Tween-20 in PBS). Primary and secondary staining were performed in 5% FBS, 0.05 M glycine in PBST. Primary antibodies used were the same as used for immunoblotting. Secondary antibodies were the Alexa Fluor series from Thermo Fisher Scientific. Nuclear chromatin staining was performed by incubation in blocking solution containing 0.5 mg/mL 4’,6-diamidino-2-phenylindole, DAPI (Sigma-Aldrich). Slides were mounted with ProLong Gold Anti-Fade Reagent (Thermo Fisher Scientific), imaged on a Leica SP8 model confocal microscope and analyzed using ImageJ. For mitochondria staining, cells were pre-stained with MitoTracker Red (Thermo Fisher Scientific) for 45 min at 37°C before starting the procedure.

### Flow cytometry

Cells were harvested, washed with PBS, and collected using an Accuri C6 CSampler (BD Biosciences). Analysis was performed using FlowJo software.

### Quantitative RT-PCR

RNA was extracted from cells using Trizol reagent (Invitrogen) according to the manufacturer’s instruction. For one-step qRT-PCR, RNA was quantified using Luna Universal One-Step qRT-PCR Kit (NEB). Analyses were performed using an Mx3000P (Stratagene).

### Statistical analyses

Results from all studies were compared with unpaired two-tailed Student’s t test, unless stated otherwise. p values less than 0.05 were considered significant.

## Supporting information

S1 Fig Multiple sequence alignment of CMPK2. Human *(Homo sapiens*), green monkey (*Chlorocebus sabaeus)*, mouse (*Mus musculus*), rat (*Rattus norvegicus)*, dog (*Canis lupus familiaris)*, and zebrafish (*Danio rerio)*. CMPK2 protein sequences were aligned using SnapGene. Identical amino acid residues (relative to human) are highlighted in yellow. The colored bars indicate amino acid conservation between species from low (blue) to high (red). Sequences of human CMPK2 (Gene ID: 129607), green monkey (103220884), mouse (22169), rat (314004), dog (608996) and zebrafish (570478) were used for the analysis.
(PDF)

S2 Fig qRT-PCR analysis. **(A)** *CMPK2* and **(B)** *RSAD2* (Viperin) RNA in Vero *i*-EV and *i-*CMPK2 cells that were mock-, doxycycline-, and type I interferon treated for 24 h or infected with Zika virus (MOI of 1) for 24 h. Data are shown as mean ± SD of two biological repeats (n = 2). Doxy = doxycycline.
(PDF)

S3 Fig Immunofluorescence analysis of Vero *i-*CMPK2 cells. Cells were doxycycline-treated for 24 h, stained with MitoTracker, fixed, permeabilized and stained by secondary only antibody (goat anti rabbit Alexa Fluor 488). Doxy = doxycycline.
(PDF)

S4 Fig Immunofluorescence analysis of the ZIKV E proteins in Vero *i*-CMPK2 cells. Cells were doxycycline-treated for 24 h then mock-infected. 48 h later, cells were fixed, permeabilized and stained by anti-E protein and anti-FLAG antibodies. Doxy = doxycycline.
(PDF)

S5 Fig mtROS production in Vero *i*-EV and *i*-CMPK2 cells. Cells were treated with doxycycline for 24 h, then incubated with MitoSOX for 10 min at 37°C, washed and analyzed by flow cytometry. MitoSOX was applied according to the manufacturing protocol (MitoSOX Red Mitochondrial Superoxide Indicator, Thermo Fisher Scientific). MitoSOX-positive cells shown in left panel and MFI shown in right panel. Antimycin A was used as a positive control. MFI = mean fluorescence intensity. Doxy = doxycycline.
(PDF)

S6 Fig Cellular concentrations of CTP nucleotide in Vero *i-*CMPK2 variant cells. Cells were doxycycline-treated for 24 h, and then collected, washed, lyophilized and resuspended in diH_2_O before high-performance liquid chromatography (HPLC) analysis [81,82]. CTP concentrations were normalized to Vero *i*-EV cell values. Data are shown as mean ± SD of two biological repeats (n = 2). ns = not significant; *p < 0.05 by one-way ANOVA.
(PDF)

S7 Fig **(A)** Immunofluorescence analysis of Vero *i-*CMPK2 cells that were doxycycline-treated for 24 h then mock-infected. 48 h later, cells were fixed, permeabilized and stained by anti-FLAG antibody for CMPK2 and J2 antibody for dsRNA detection. Doxy = doxycycline. **(B)** The CellTiter-Glo (CTG) Luminescent Cell Viability Assay. ATP production in doxycycline-treated Vero *i*-EV and *i*-CMPK2 variant cells upon NanoLuc luciferase reporter ZIKV infection (MOI = 0.5). At indicated time post infection, the luminescent signal was measured according to the manufacturing protocol (CellTiter-Glo Luminescent, Promega). **(C)** Luciferase activity assay in Vero *i*-EV cells that were electroporated with the *in vitro* transcribed ZIKV WT and/or NS5 GAA mutant RNA encoding a luciferase reporter ZIKV. At indicated time post electroporation, the cells were lysed and measured for luciferase activities. **(D)** qRT-PCR analysis of virus entry assay which was performed as reported by Le Sommer *et al*. [54]. Vero *i-*EV and -CMPK2 cells were doxycycline-treated for 24 h, then incubated with ZIKV at an MOI of 5 at 37°C for 2 h. Uninternalized virus particles were removed by washing the cells twice with cold PBS, followed by a 3-min exposure to 1 M NaCl and 50 mM Na_2_CO_3_, pH 9.5. **(E)** Vero *i-*CMPK2 cells were mock- or doxycycline-treated for 24 h, then incubated with ZIKV labeled with DiOC18 at 4°C at an MOI of 2 for 30 min [55]. After 30 min the cells were washed and collected. Additional samples were incubated at 37°C for 1 h, in the presence or absence of NH_4_Cl to block acidification. Then washed, fixed and analyzed by flow cytometry (% of DiOC18-positive cells shown in left panel and MFI shown in right panel). MFI = mean fluorescence intensity. Doxy = doxycycline.
(PDF)

S8 Fig Global protein synthesis. **(A)** Luciferase activity assay in Vero *i*-EV and *i*-CMPK2 cells that were doxycycline-treated for 24 h then transfected with *in vitro* transcribed Firefly luciferase (Fluc) reporter mRNA (CleanCap Fluc mRNA (L-7602), TriLink) [83]. At indicated time post transfection, the cells were lysed and measured for luciferase activities. **(B)** OP-Puro labeling in 293T cells transfected with EV control and CMPK2 WT for 24 h, and analyzed by flow cytometry [12,58]. MFI = mean fluorescence intensity; CHX = cycloheximide.
(PDF)

S1 Table Primer list. (PDF)
