# Supplementary material for: CMPK2 restricts Zika virus replication by inhibiting viral translation
Source: PLoS Pathog. 2023 Apr 19;19(4):e1011286. doi: 10.1371/journal.ppat.1011286 (PMC10150978; doi:10.1371/journal.ppat.1011286)
Supplement: S1 Fig — Human (Homo sapiens), green monkey (Chlorocebus sabaeus), mouse (Mus musculus), rat (Rattus norvegicus), dog (Canis lupus familiaris), and zebrafish (Danio rerio). CMPK2 protein sequences were aligned using SnapGene. Identical amino acid residues (relative to human) are highlighted in yellow. The colored bars indicate amino acid conservation between species from low (blue) to high (red). Sequences of human CMPK2 (Gene ID: 129607), green monkey (103220884), mouse (22169), rat (314004), dog (608996) and zebrafish (570478) were used for the analysis. (PDF) [file ppat.1011286.s001.pdf]

Degree of conservation:

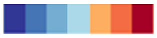

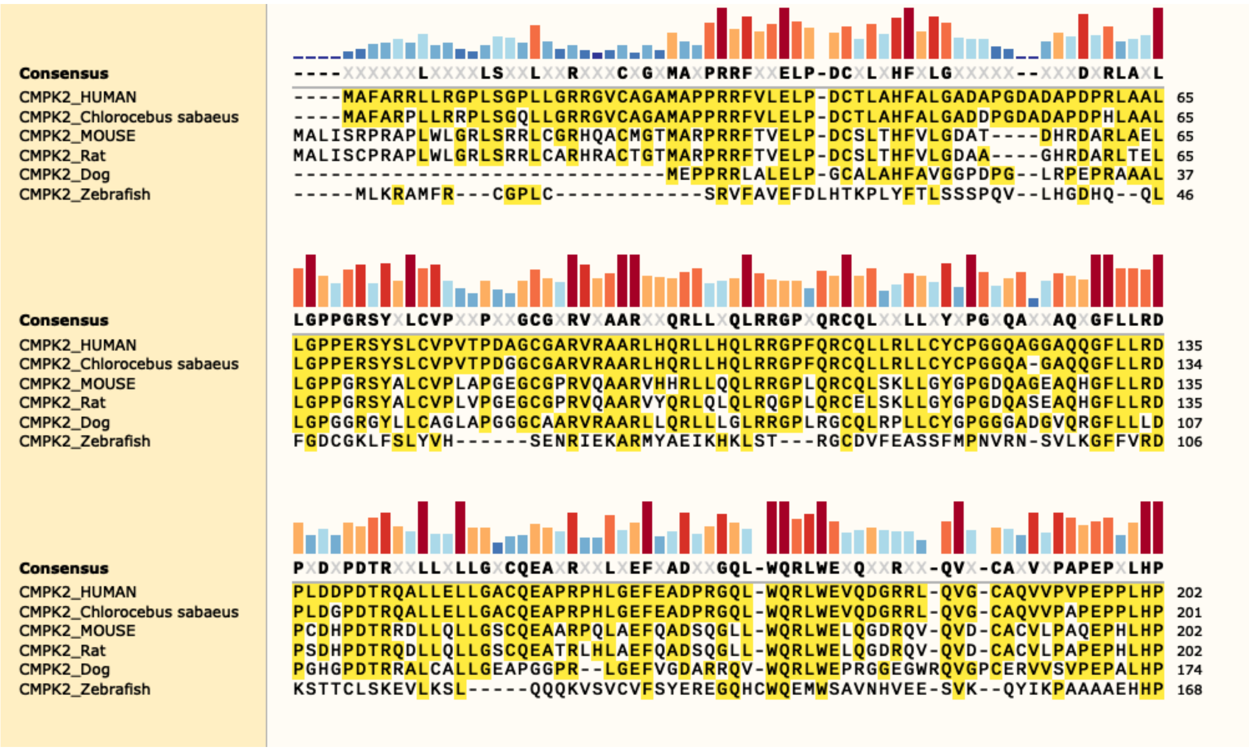

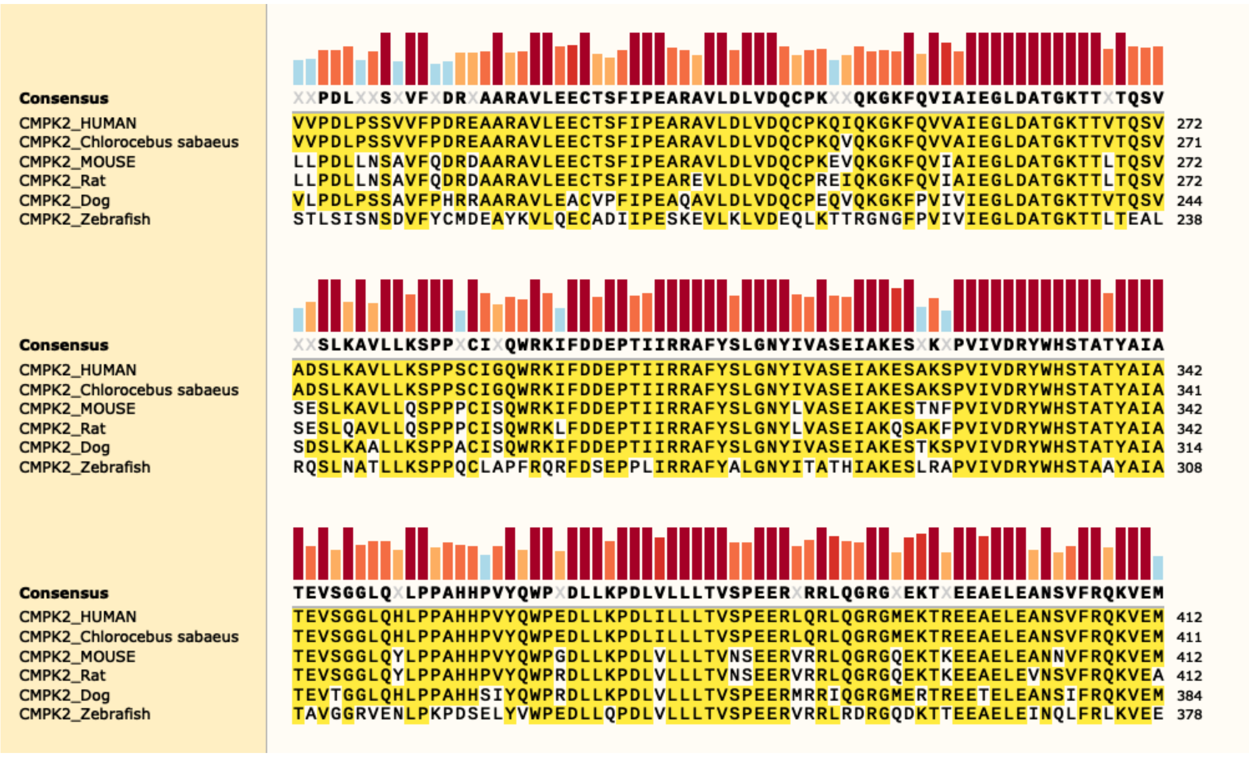

S1 Fig. Multiple sequence alignment of CMPK2.

Human *(Homo sapiens*), green monkey (*Chlorocebus sabaeus)*, mouse (*Mus musculus*), rat (*Rattus norvegicus)*, dog (*Canis lupus familiaris)*, and zebrafish (*Danio rerio).* CMPK2 protein sequences were aligned using SnapGene. Identical amino acid residues (relative to human) are highlighted in yellow. The colored bars indicate amino acid conservation between species from low (blue) to high (red). Sequences of human CMPK2 (Gene ID: 129607), green monkey (103220884), mouse (22169), rat (314004), dog (608996) and zebrafish (570478) were used for the analysis.
