# Supplementary material for: CMPK2 restricts Zika virus replication by inhibiting viral translation
Source: PLoS Pathog. 2023 Apr 19;19(4):e1011286. doi: 10.1371/journal.ppat.1011286 (PMC10150978; doi:10.1371/journal.ppat.1011286)
Supplement: S2 Fig — (A) CMPK2 and (B) RSAD2 (Viperin) RNA in Vero i-EV and i-CMPK2 cells that were mock-, doxycycline-, and type I interferon treated for 24 h or infected with Zika virus (MOI of 1) for 24 h. Data are shown as mean ± SD of two biological repeats (n = 2). Doxy = doxycycline. (PDF) [file ppat.1011286.s002.pdf]

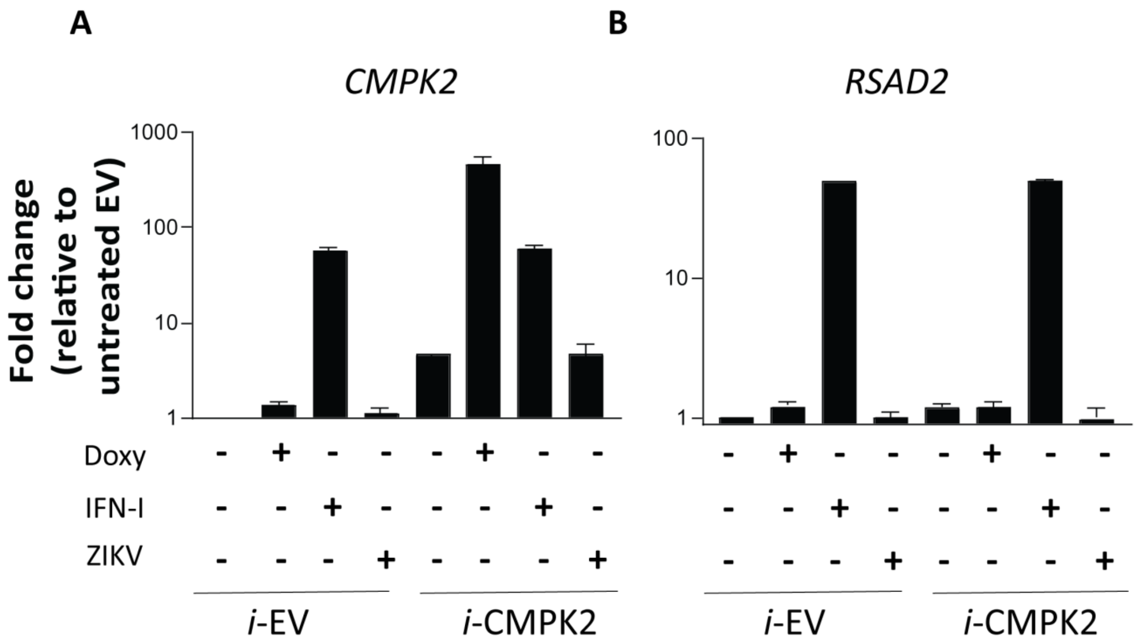

S2 Fig. qRT-PCR analysis. (A) *CMPK2* and **(B)** *RSAD2* (Viperin) RNA in Vero *i*-EV and *i*-CMPK2 cells that were mock-, doxycycline-, and type I interferon treated for 24 h or infected with Zika virus (MOI of 1) for 24 h. Data are shown as mean ± SD of two biological repeats (n = 2). Doxy = doxycycline.
