# Supplementary material for: CMPK2 restricts Zika virus replication by inhibiting viral translation
Source: PLoS Pathog. 2023 Apr 19;19(4):e1011286. doi: 10.1371/journal.ppat.1011286 (PMC10150978; doi:10.1371/journal.ppat.1011286)
Supplement: S3 Fig — Cells were doxycycline-treated for 24 h, stained with MitoTracker, fixed, permeabilized and stained by secondary only antibody (goat anti rabbit Alexa Fluor 488). Doxy = doxycycline. (PDF) [file ppat.1011286.s003.pdf]

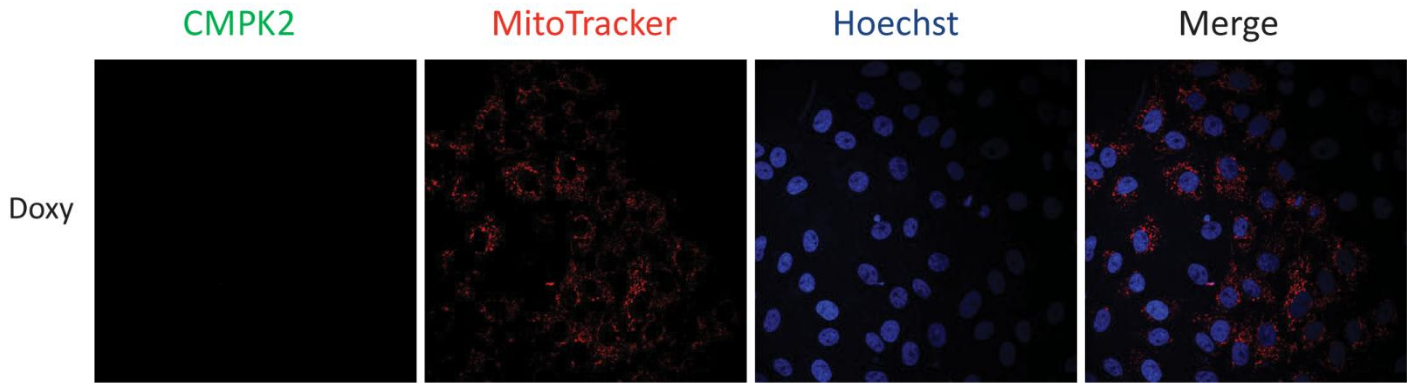

S3 Fig. Immunofluorescence analysis of Vero *i*-CMPK2 cells. Cells were doxycycline-treated for 24 h, stained with MitoTracker, fixed, permeabilized and stained by secondary only antibody (goat anti rabbit Alexa Fluor 488). Doxy = doxycycline.
