# Supplementary material for: CMPK2 restricts Zika virus replication by inhibiting viral translation
Source: PLoS Pathog. 2023 Apr 19;19(4):e1011286. doi: 10.1371/journal.ppat.1011286 (PMC10150978; doi:10.1371/journal.ppat.1011286)
Supplement: S4 Fig — Cells were doxycycline-treated for 24 h then mock-infected. 48 h later, cells were fixed, permeabilized and stained by anti-E protein and anti-FLAG antibodies. Doxy = doxycycline. (PDF) [file ppat.1011286.s004.pdf]

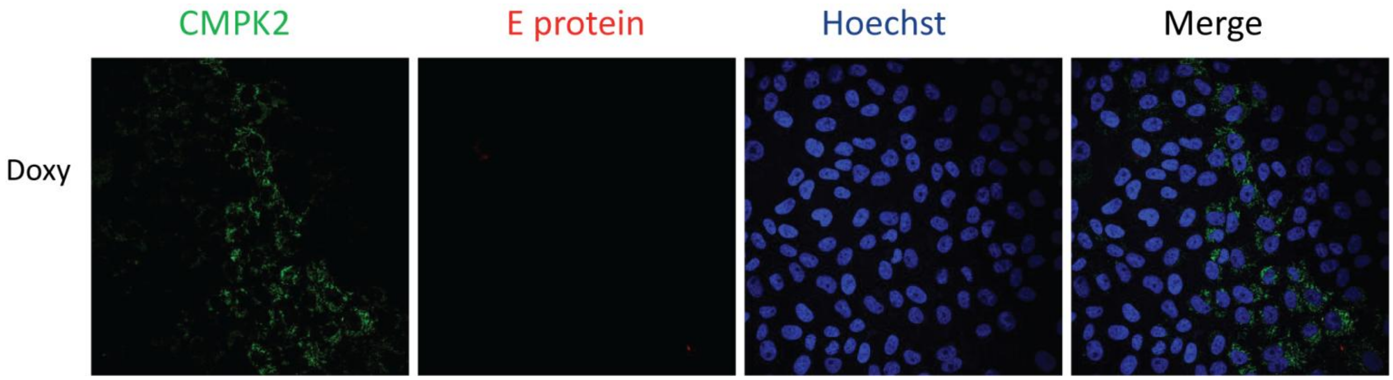

S4 Fig. Immunofluorescence analysis of the ZIKV E proteins in Vero *i*-CMPK2 cells. Cells were doxycycline-treated for 24 h then mock-infected. 48 h later, cells were fixed, permeabilized and stained by anti-E protein and anti-FLAG antibodies. Doxy = doxycycline.
