# Supplementary material for: CMPK2 restricts Zika virus replication by inhibiting viral translation
Source: PLoS Pathog. 2023 Apr 19;19(4):e1011286. doi: 10.1371/journal.ppat.1011286 (PMC10150978; doi:10.1371/journal.ppat.1011286)
Supplement: S5 Fig — Cells were treated with doxycycline for 24 h, then incubated with MitoSOX for 10 min at 37°C, washed and analyzed by flow cytometry. MitoSOX was applied according to the manufacturing protocol (MitoSOX Red Mitochondrial Superoxide Indicator, Thermo Fisher Scientific). MitoSOX-positive cells shown in left panel and MFI shown in right panel. Antimycin A was used as a positive control. MFI = mean fluorescence intensity. Doxy = doxycycline. (PDF) [file ppat.1011286.s005.pdf]

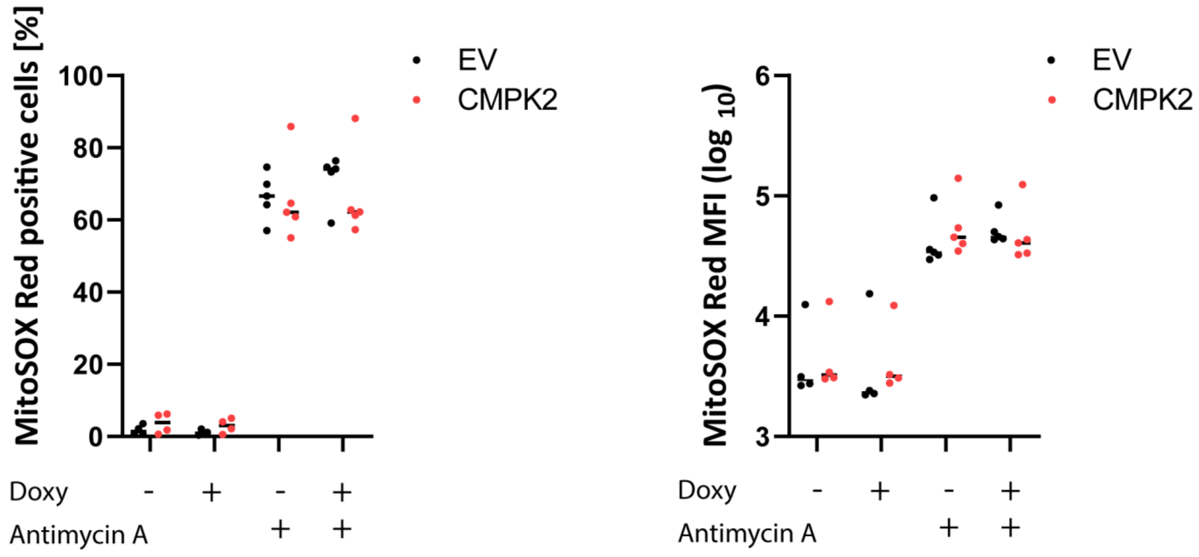

S5 Fig. mtROS production in Vero *i*-EV and *i*-CMPK2 cells. Cells were treated with doxycycline for 24 h, then incubated with MitoSOX for 10 min at 37˚C, washed and analyzed by flow cytometry. MitoSOX was applied according to the manufacturing protocol (MitoSOX Red Mitochondrial Superoxide Indicator, Thermo Fisher Scientific). MitoSOX-positive cells shown in left panel and MFI shown in right panel. Antimycin A was used as a positive control. MFI = mean fluorescence intensity. Doxy = doxycycline.
