# Supplementary material for: CMPK2 restricts Zika virus replication by inhibiting viral translation
Source: PLoS Pathog. 2023 Apr 19;19(4):e1011286. doi: 10.1371/journal.ppat.1011286 (PMC10150978; doi:10.1371/journal.ppat.1011286)
Supplement: S6 Fig — Cells were doxycycline-treated for 24 h, and then collected, washed, lyophilized and resuspended in diH2O before high-performance liquid chromatography (HPLC) analysis [81,82]. CTP concentrations were normalized to Vero i-EV cell values. Data are shown as mean ± SD of two biological repeats (n = 2). ns = not significant; *p < 0.05 by one-way ANOVA. (PDF) [file ppat.1011286.s006.pdf]

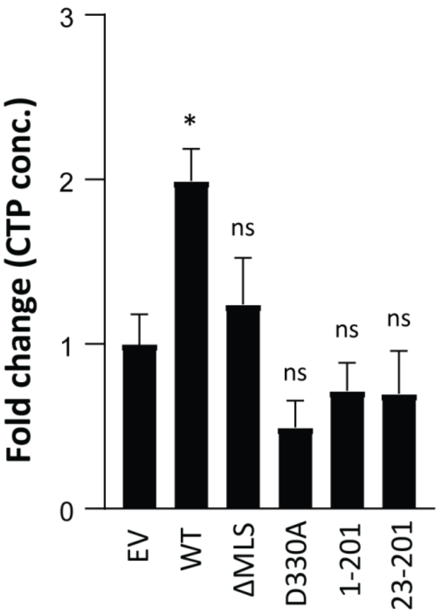

S6 Fig. Cellular concentrations of CTP nucleotide in Vero *i*-CMPK2 variant cells. Cells were doxycycline-treated for 24 h, and then collected, washed, lyophilized and resuspended in diH_2O before high-performance liquid chromatography (HPLC) analysis. CTP concentrations were normalized to Vero *i*-EV cell values. Data are shown as mean ± SD of two biological repeats (n = 2). ns = not significant; *$p < 0.05$ by one-way ANOVA.
