# Supplementary material for: CMPK2 restricts Zika virus replication by inhibiting viral translation
Source: PLoS Pathog. 2023 Apr 19;19(4):e1011286. doi: 10.1371/journal.ppat.1011286 (PMC10150978; doi:10.1371/journal.ppat.1011286)
Supplement: S7 Fig — (A) Immunofluorescence analysis of Vero i-CMPK2 cells that were doxycycline-treated for 24 h then mock-infected. 48 h later, cells were fixed, permeabilized and stained by anti-FLAG antibody for CMPK2 and J2 antibody for dsRNA detection. Doxy = doxycycline. (B) The CellTiter-Glo (CTG) Luminescent Cell Viability Assay. ATP production in doxycycline-treated Vero i-EV and i-CMPK2 variant cells upon NanoLuc luciferase reporter ZIKV infection (MOI = 0.5). At indicated time post infection, the luminescent signal was measured according to the manufacturing protocol (CellTiter-Glo Luminescent, Promega). (C) Luciferase activity assay in Vero i-EV cells that were electroporated with the in vitro transcribed ZIKV WT and/or NS5 GAA mutant RNA encoding a luciferase reporter ZIKV. At indicated time post electroporation, the cells were lysed and measured for luciferase activities. (D) qRT-PCR analysis of virus entry assay which was performed as reported by Le Sommer et al. [54]. Vero i-EV and -CMPK2 cells were doxycycline-treated for 24 h, then incubated with ZIKV at an MOI of 5 at 37°C for 2 h. Uninternalized virus particles were removed by washing the cells twice with cold PBS, followed by a 3-min exposure to 1 M NaCl and 50 mM Na2CO3, pH 9.5. (E) Vero i-CMPK2 cells were mock- or doxycycline-treated for 24 h, then incubated with ZIKV labeled with DiOC18 at 4°C at an MOI of 2 for 30 min [55]. After 30 min the cells were washed and collected. Additional samples were incubated at 37°C for 1 h, in the presence or absence of NH4Cl to block acidification. Then washed, fixed and analyzed by flow cytometry (% of DiOC18-positive cells shown in left panel and MFI shown in right panel). MFI = mean fluorescence intensity. Doxy = doxycycline. (PDF) [file ppat.1011286.s007.pdf]

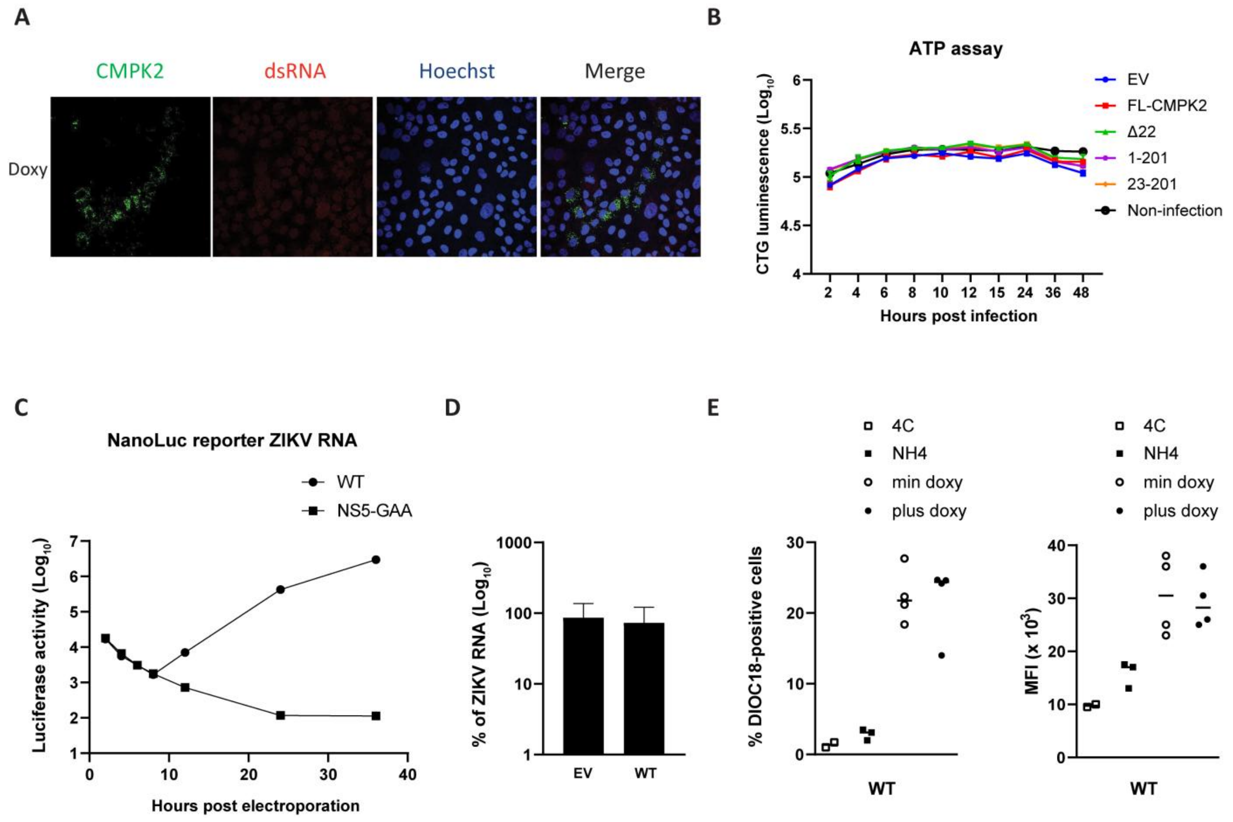

S7 Fig. (A) Immunofluorescence analysis of Vero *i*-CMPK2 cells that were doxycycline-treated for 24 h then mock-infected. 48 h later, cells were fixed, permeabilized and stained by anti-FLAG antibody for CMPK2 and J2 antibody for dsRNA detection. Doxy = doxycycline.

(B) The CellTiter-Glo (CTG) Luminescent Cell Viability Assay. ATP production in doxycycline-treated Vero *i*-EV and *i*-CMPK2 variant cells upon NanoLuc luciferase reporter ZIKV infection (MOI = 0.5). At indicated time post infection, the luminescent signal was measured according to the manufacturing protocol (CellTiter-GloLuminescent, Promega).

(C) Luciferase activity assay in Vero *i*-EV cells that were electroporated with the *in vitro* transcribed ZIKV WT and NS5 GAA mutant RNA encoding a luciferase reporter ZIKV. At indicated time post electroporation, the cells were lysed and measured for luciferase activities.

(D) qRT-PCR analysis of virus entry assay which was performed as reported by Le Sommer *et al*. Vero *i*-EV and -CMPK2 cells were doxycycline-treated for 24 h, then incubated with ZIKV at an MOI of 5 at 37°C for 2 h. Uninternalized virus particles were removed by washing the cells twice with cold PBS, followed by a 3-min exposure to 1 M NaCl and 50 mM Na_2CO_3, pH 9.5.

(E) Vero *i*-CMPK2 cells were mock- or doxycycline-treated for 24 h, then incubated with ZIKV labeled with DiOC18 at 4°C at an MOI of 2 for 30 min. After 30 min the cells were washed and collected. Additional samples were incubated at 37°C for 1 h, in the presence or absence of NH_4Cl to block acidification. Then washed, fixed and analyzed by flow cytometry (% of DiOC18-positive cells shown in left panel and MFI shown in right panel). MFI = mean fluorescence intensity. Doxy = doxycycline.
