# Supplementary material for: CMPK2 restricts Zika virus replication by inhibiting viral translation
Source: PLoS Pathog. 2023 Apr 19;19(4):e1011286. doi: 10.1371/journal.ppat.1011286 (PMC10150978; doi:10.1371/journal.ppat.1011286)
Supplement: S8 Fig — (A) Luciferase activity assay in Vero i-EV and i-CMPK2 cells that were doxycycline-treated for 24 h then transfected with in vitro transcribed Firefly luciferase (Fluc) reporter mRNA (CleanCap Fluc mRNA (L-7602), TriLink) [83]. At indicated time post transfection, the cells were lysed and measured for luciferase activities. (B) OP-Puro labeling in 293T cells transfected with EV control and CMPK2 WT for 24 h, and analyzed by flow cytometry [12,58]. MFI = mean fluorescence intensity; CHX = cycloheximide. (PDF) [file ppat.1011286.s008.pdf]

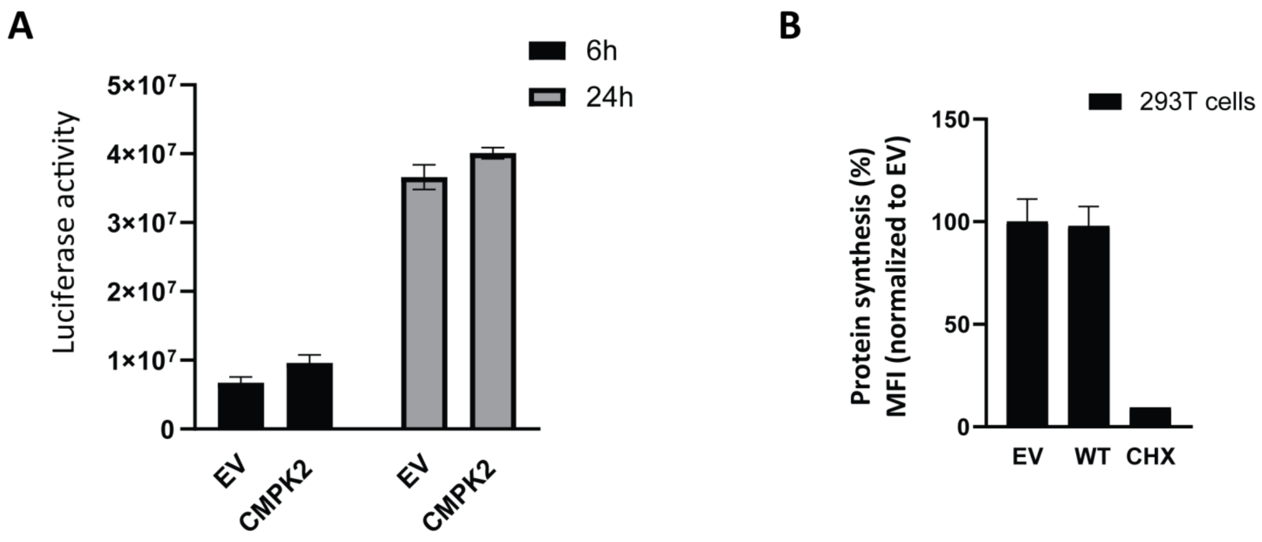

S8 Fig. Global protein synthesis. (A) Luciferase activity assay in Vero *i*-EV and *i*-CMPK2 cells that were doxycycline-treated for 24 h then transfected with *in vitro* transcribed Firefly luciferase (Fluc) reporter mRNA (CleanCap Fluc mRNA (L-7602), TriLink). At indicated time post transfection, the cells were lysed and measured for luciferase activities.

(B) OP-Puro labeling in 293T cells transfected with EV control and CMPK2 WT for 24 h, and analyzed by flow cytometry. MFI = mean fluorescence intensity. CHX = cycloheximide.
