# Supplementary material for: CMPK2 restricts Zika virus replication by inhibiting viral translation
Source: PLoS Pathog. 2023 Apr 19;19(4):e1011286. doi: 10.1371/journal.ppat.1011286 (PMC10150978; doi:10.1371/journal.ppat.1011286)
Supplement: S1 Table — (PDF) [file ppat.1011286.s009.pdf]

S1 Table. Primer list.

Primer no.	Gene	Primer sequence	Reason
1	RSAD2	**F:** CTGAAGCTGGATTGGTAGAG	**qRT-PCR**
2		**R:** GGGAAATACCAACGGGATAG	**qRT-PCR**
3	CMPK2	**F:** CCAGGTTGTTGCCATCGAAG	**qRT-PCR**
4		**R:** CAAGAGGGTGGTGACTTTAAGAG	**qRT-PCR**
5		**F:** CACCGAGCGAGTAGCTGCGCTCCGG	**gRNA**
6		**R:** AAACCCGGAGCGCAGCTACTCGCTC	**gRNA**
7		**F:** CACCGCTCCTCCAAAACGGCCCGGG	**gRNA**
8		**R:** AAACCCCGGGCCGTTTTGGAGGAGC	**gRNA**
9	GAPDH	**F:** ACAACTTTGGTATCGTGGAAGG	**qRT-PCR**
10		**R:** GCCATCACGCCACAGTTTC	**qRT-PCR**
